# Supplementary material for: Preventing depression in the community by voluntary sector providers (PERSUADE): intervention development and protocol for a parallel randomised controlled feasibility trial
Source: BMJ Open. 2019 Oct 3;9(10):e023791. doi: 10.1136/bmjopen-2018-023791 (PMC6797240; doi:10.1136/bmjopen-2018-023791)
Supplement: Author's manuscript [file bmjopen-2018-023791.draft_revisions.pdf]

BMJ Open is committed to open peer review. As part of this commitment we make the peer review history of every article we publish publicly available.

When an article is published we post the peer reviewers' comments and the authors' responses online. We also post the versions of the paper that were used during peer review. These are the versions that the peer review comments apply to.

The versions of the paper that follow are the versions that were submitted during the peer review process. They are not the versions of record or the final published versions. They should not be cited or distributed as the published version of this manuscript.

BMJ Open is an open access journal and the full, final, typeset and author-corrected version of record of the manuscript is available on our site with no access controls, subscription charges or pay-per-view fees (http://bmjopen.bmj.com).

If you have any questions on BMJ Open's open peer review process please email info.bmjopen@bmj.com

BMJ Open

Preventing depression in the community by voluntary sector providers (PERSUADE): intervention development and protocol for a pilot randomised controlled trial.

Journal:	*BMJ Open*
Manuscript ID	bmjopen-2018-023791
Article Type:	Protocol
Date Submitted by the Author:	25-Apr-2018

Complete List of Authors:	Kenning, Cassandra; University of Manchester, Division of Population Health, Health Services Research and Primary Care<br>Blakemore, Amy; University of Manchester,<br>Bower, Peter; University of Manchester, Division of Population Health, Health Services Research and Primary Care<br>Safari, Melina; University of Manchester, Division of Population Health, Health Services Research and Primary Care<br>Cuijpers, Pim; Vrije Universiteit Amsterdam<br>Brown, June S. L.; Kings College London<br>Hann , Mark ; University of Manchester, Centre for Biostatistics and Manchester Academic Health Science Centre<br>Lidbetter, Nicky; Self Help Services<br>Muñoz, Ricardo; Palo Alto University<br>Waheed, Waquas; University of Manchester, Division of Population Health, Health Services Research and Primary Care

Keywords:	Depression < mood disorders < PSYCHIATRY, PUBLIC HEALTH, PREVENTIVE MEDICINE, MENTAL HEALTH

SCHOLARONE™ Manuscripts

Preventing depression in the community by voluntary sector providers (PERSUADE): intervention development and protocol for a pilot randomised controlled trial.

Cassandra Kenning. Division of Population Health, Health Services Research & Primary Care. The University of Manchester, Manchester UK.

Amy Blakemore. Division of Nursing, Social Work & Midwifery. The University of Manchester, Manchester UK.

Peter Bower. Division of Population Health, Health Services Research & Primary Care. The University of Manchester, Manchester UK.

Melina Safari. Division of Population Health, Health Services Research & Primary Care. The University of Manchester, Manchester UK.

Pim Cuijpers, Faculty of Behaviour and Movement Sciences. Vrije Universiteit, Amsterdam, The Netherlands.

June Brown, Department of Psychology. King's College London, London UK.

Mark Hann. Centre for Biostatistics and Manchester Academic Health Science Centre. The University of Manchester, Manchester UK.

Nicky Lidbetter, Self Help Services, Manchester UK.

Ricardo Muñoz, Clinical Psychology. Palo Alto University. Palo Alto CA, US.

Waquas Waheed* Division of Population Health, Health Services Research & Primary Care. The University of Manchester, Manchester UK.

*corresponding author

Dr Waquas Waheed

The University of Manchester

5th Floor Williamson Building

Oxford Road

Manchester

M13 9PL

Waquas.waheed@manchester.ac.uk

Tel: 0161 306 7949

Word count: 2733

For peer review only - http://bmjopen.bmj.com/site/about/guidelines.xhtml

Abstract

Introduction

Depression is now the most common illness worldwide affecting more than 300,000 people. Yet studies modelling the impact of depression interventions have shown that the burden of depression cannot be minimised by more than 35% with existing treatments. There is a need to develop better preventative interventions. The overall aim of this programme of work is to develop interventions to reduce depression symptom scores and prevent development of depression in people with sub-threshold depression. The objectives are to adapt a low intensity community based depression prevention intervention, establish the acceptability and uptake of this model, and to conduct a small pilot study to inform a full-scale trial.

Methods and analysis

A series of focus groups will be run with members of the public, charity and voluntary sector providers, and researchers to inform the adaptation of an existing eight week depression prevention course. Sixty four people with sub-threshold depression, as represented by a score of between 5 and 9 on the PHQ-9 depression measure, will be recruited to take part in a pilot trial. Participants will be randomised equally to the intervention or usual care control groups. Participants in the intervention group will receive the new developed manual and attend a one-day workshop delivered by a voluntary sector service provider. Outcome measures will be completed three months after baseline. Quantitative data on recruitment, randomisation, attendance, retention, questionnaire completion rates will be collected. Primary analyses will be descriptive. Interviews will be conducted to explore acceptability and framework analysis will be used to analyse the data.

Ethics and dissemination

The study has been reviewed and approved by NHS Research and Ethics Committee: NW-Greater Manchester East. The results will be actively disseminated through peer-reviewed journals, conference presentations, social media, the internet and community engagement activities.

Trial registration: ISRCTN23278208

Key words: sub-threshold, depression, prevention, protocol, pilot RCT.

For peer review only - http://bmjopen.bmj.com/site/about/guidelines.xhtml

Strengths and limitations of this study

- By adapting the intervention to a workbook and one-day workshop this will reduce costs, make it more accessible (lower burden) to participants, and move the intervention from primary care to voluntary sector organisations.
- Recruiting people with sub-threshold depression can be challenging and so the study will evaluate a number of different recruitment strategies and assess them to see how much time each takes and the associated yield.
- We are engaging substantively with members of the public as well as with voluntary sector services and charities to ensure the intervention is both feasible and acceptable.
- This is a pilot feasibility study and is not powered to show effectiveness of the intervention.

For peer review only

For peer review only - http://bmjopen.bmj.com/site/about/guidelines.xhtml

Introduction

Depression has high prevalence and incidence,[1] affects quality of life,[2] productivity, fulfilment of social roles, and increases mortality,[3, 4] leading to high service use and economic costs.[5] Depression is a major contributor to global disease burden, predicted to become the leading contributor in high-income countries by 2030.[6] Recent reports by the World Health Organisation (WHO), show that depression is now the most common illness worldwide affecting more than 300,000 people.[7] In a recent community cohort study of whites and British Pakistanis, prevalence of depression was 12.6%. At 6 months follow up 60.5% cases were persistently depressed and incidence of new cases was 17%.[8]

Modelling studies have shown that existing treatments cannot reduce the burden of depression by more than 35%, even under ideal circumstances.[9] Prevention may offer an opportunity to reduce the disease burden of depression further.[10] A number of studies have examined the capability of prevention programmes to successfully reduce the incidence of depression.[11] This is important from a public health perspective as duration of the disorder is inversely related to outcome, so that by the time cases are recognised, they are harder to treat. Developing an intervention aimed at both prevention and early treatment could potentially reduce the overall burden of depression.[6]

NICE guidelines (CG 90 and 91) advocate case-finding by GP for depression in high risk groups with physical comorbidities, and those with past history of depression. They recommend offering anti-depressants or psychological interventions to people with moderate to severe depression.[12] Less attention has been paid to sub-threshold depression, defined as those who respond positively to case-finding questions, such as on the Patient Health Questionnaire (PHQ-9)[13] but do not have sufficient symptom scores on diagnostic criteria.[14] Sub-threshold depression is a major risk factor for progression and development of more severe depression[15, 16] and has been shown to have similar consequences.[17-19]

Previous UK trials have generally included cases with above threshold depression and have not looked exclusively at people with sub-threshold depression. We could identify only 2 UK RCTs in the two published meta-analyses of 25 depression prevention trials.[20, 21] Both UK trials were aimed exclusively at pregnant women and published in 2000. An update of this meta-analysis included 34 RCTs with no further RCTs from UK.[22]

An intervention for sub-threshold depression needs to reduce the incidence of new onset depression but also improve outcome for those who do develop depression by reducing the length of the episode. GPs coordinating with voluntary sector organisations to deliver the intervention will potentially increase access for hard to reach groups.[23-25] However, voluntary sector workers will need to be adequately trained and supervised in delivering a patient-centred intervention according to a treatment manual.

We need to understand user and provider (GP and therapists) views about prevention strategies in subthreshold depression and their applicability to UK health systems. There is uncertainty around the content, format, duration and use of voluntary sector workers to deliver such an intervention. We will aim to address these issues through involving users, providers, primary care staff and experts in this field and incorporating their opinions into the intervention and trial design.

For peer review only - http://bmjopen.bmj.com/site/about/guidelines.xhtml

The model we propose to use will be a psycho-educational intervention for prevention of depression in the form of a community based workshop to be delivered to people with sub-threshold depression. The proposed intervention will be adapted from the "Depression Prevention Course" and include evidence based CBT techniques, previously developed by Muñoz for preventing depression.[26] The adapted intervention will be delivered via an accessible community workshop format as developed for depressed people by Brown (Self-confidence workshops).[24, 25, 27]

Research objectives

The eventual aim of our research programme is to reduce depression symptom scores and prevent development of depression in people with sub-threshold depression defined as PHQ score 5-9.[13] The feasibility study will follow the MRC framework[28] for the development and evaluation of complex interventions, and has the following objectives:

1. To adapt a low intensity community based depression prevention intervention based on CBT model for adults with sub-threshold depression deliverable by voluntary sector organisations.

2. To establish the acceptability and uptake of this model by adults with sub-threshold depression.

3. To test the feasibility of conducting a successful trial of community based depression prevention intervention for adults with sub-threshold depression.

Methods and analysis

Patient and Public Involvement

Over a number of years we have conducted research in collaboration with community based voluntary organisations (Roby Church, Mind, Pakistani resource centre, Scaitcliffe Community Centre) in the North West. During past stakeholder events it had been discussed that interventions should be made available that prevent depression in vulnerable people.

During the preparation of the funding application we worked with voluntary sector organisations and providers (YARAN Community services, Self Help Services, Manchester) to inform the proposed intervention content, delivery and research design. We also made presentations to a lay public group and incorporated their suggestions into the proposal. YARAN Community services and Self Help Services, Manchester will offer advice throughout the project. Phase 1 of the study involves community focus groups and at the end of the study community events will be used to feed back the results and to develop the proposal for a full trial for effectiveness and cost-effectiveness.

Qualitative interviews with participants in the intervention group will focus on their experiences of a, taking part in a study and b, all aspects of the intervention (manual and workshop) to explore acceptability and to further modify or adapt the intervention and trial design for a full-trial.

For peer review only - http://bmjopen.bmj.com/site/about/guidelines.xhtml

Phase 1- intervention development

Intervention and manual development: The 'Depression Prevention Course' developed by Muñoz[26] will be adapted for this study.

Box 1- Depression Prevention Course

Originally based on Social Learning Theory<br><br>The intervention was developed using materials from the book 'Control your Depression' 1986<br><br>Combines three methods used to treat depression:<br>• Increasing pleasant activities<br>• Social skills training<br>• Cognitive approaches<br><br>Delivered by trained CBT therapist<br><br>8 group sessions delivered over 16 weeks<br><br>51 page 'Outlines for participants' manual (text only)

Focus groups: To tailor the intervention, focus groups will be held with users and providers.

A series of focus groups (3-5) will be run with a variety of groups including the general population, voluntary service providers from Self-help Manchester, and academic participants. The aim of the groups will be to get feedback on intervention content, delivery options, manual design, as well as recruitment methods. This will assist in the intervention adaptation and delivery, and also in refining the recruitment methods and materials. The groups will be held at a location convenient to participants and last around 2 hours. Sessions will be audio recorded, with consent, and transcribed.

Prevent depression Intervention:

Once we have run the focus groups, and discussed the outcomes within the trial team, the original Muñoz manual will be redesigned as an accessible and easy to understand manual based intervention. To support the manual and guide people in its use, participants will attend a one day (or two half-day) workshop. The aim is that offering a one-off psychoeducational group will improve access for a larger group of people. Asking participants to commit to 16 weeks of intervention when they do not yet have a diagnosable condition requires substantial resources and time commitment from service providers and participants. This workshop approach has been effective in a number of studies.[23-27]

Phase 2- pilot study

For peer review only - http://bmjopen.bmj.com/site/about/guidelines.xhtml

PERSUADE Protocol v2 (20/08/2017)

Design: A mixed methods pilot study and process evaluation to explore feasibility and acceptability of a depression prevention intervention.

Setting: Primary care and community, Greater Manchester UK.

Inclusion criteria: Patients from primary care and the general community who are aged above 18 years and who score between 5 and 9 on the PHQ 9.[13]

Exclusion criteria:

- Depression (score higher than 9 on PHQ-9, or formal diagnosis of depression)
- Psychosis (self-report)
- Drug or alcohol use as primary diagnosis (self-report)
- Suicidal ideations (self-report)
- Non-English language speakers

Participant recruitment: As this is a feasibility study, a formal sample size calculation was not undertaken. The aim is to recruit a total of 64 patients to be randomised with equal probability to the intervention or usual care (i.e. 32 to each).

Considering the challenge of recruiting persons with sub-threshold depression, a number of different recruitment strategies will be tried and assessed to see how much time each takes and the associated yield.

Recruitment through GP practices:

- GPs to refer patients recently screened with the PHQ-9 who scored between 5 and 9. They will either give out the study information during the consultation or post the patient materials to them.
- Searches will be carried out on GP practice databases to see if it is feasible to identify patients who have concerns about their mood but who have not been referred on to IAPT services due to scores below 9 on the PHQ-9. Searches will be done by practice staff or CRN support staff to identify potential participants and mail out the study information.
- Research staff to offer study information and to screen patients with PHQ-9 in GP waiting rooms if they are interested in taking part.

Network support will be sought from Greater Manchester NIHR Clinical Research Network (CRN) to ensure that the research project is conducted within the proposed timeframe and recruitment targets are achieved. CRN will help with engaging primary care and liaising with GP at site.

Recruitment through advertising:

- Voluntary sector organisations (Scaitcliffe centre, YARAAN community, Self-help Services, Manchester) to direct self-referring patients by providing information about the study.
- Advertise in GP practice, library, community centres, citizens' advice bureau, Sure Start and places of worship.

For peer review only - http://bmjopen.bmj.com/site/about/guidelines.xhtml

- Advertise on dedicated Website, Facebook and Twitter
- Advertise the study in local newspapers/radio.

Recruitment through other trials:

- Invite participants who are screened out from any ongoing depression trials run under Clinical Research Network. For those trials that agree, once patients have been screened as ineligible from other depression trials due to a low score on PHQ-9, they will send the details of PERSUADE trial to ineligible patients.

Consent

Patients consenting to be contacted by the research team will be contacted over the phone or if needed, visited at home or a convenient location. They will be given a participant information sheet and have the study clearly explained to them, and will be given the opportunity to ask questions. The researcher will screen the participant for eligibility (as above) and if patients agree and meet the inclusion criteria they will be asked to sign and return the 'participant consent form' to enter the trial. Baseline assessments will then be completed by questionnaire or by telephone.

Confidentiality and data storage

All participants will be assigned a unique identification number which will be used for randomisation, data collection documents and analysis. Consent forms will be stored separately from participant data in locked filing cabinets. Publication of results will retain identification numbers to preserve anonymity, particulary where direct quotes are used. Only the primary research team will have access to the final trial data set.

Ineligible and non-recruited participants

In cases where either moderate to severe depression or risk of self-harm are detected, potential participants will be referred back to their GP.

Randomisation Participants will be randomised with equal probability to the CBT-based workshops or usual care (i.e. 32 to each). Computer generated block randomisation will be used. The allocation sequence will be set up by someone independent of the research team. Once a participant is randomised the trial Research Associate will be informed by email and they will then contact participants. Participants will be informed of the allocation by letter or telephone. As this is an active intervention they will not be blinded. Also as this is a pilot study and not a trial of efficacy the research associate/PhD student will not be blind to allocation.

The figure below describes the process for the entry of participants into the trial and data collection points.

Figure 1 Recruitment and inclusion diagram * Insert here*

For peer review only - http://bmjopen.bmj.com/site/about/guidelines.xhtml

Intervention

Format and content of the intervention

We will train voluntary sector workers to facilitate community workshops that will last up to 8 hours and will be delivered either on a single day or be split over 2 days (evenings and weekends). Participants will attend one of these workshops. There will be no restrictions on the type of care participants can receive from other services.

Data on content, design and format of prevention interventions has already been extracted and will be published as a review, this will guide us in adapting the "Depression Prevention Course"[26] for this study. Motivational techniques will be incorporated in the beginning of the workshop to help engage participants, based on previous research and our own experience of conducting social groups. Group exercises, role-play demonstrations and discussions of case vignettes will be participatory to help maintain participant's interest and motivation throughout the workshop. Participants will receive a professionally designed manual, in colour and with graphics, that describes the workshop's program and all content. Workshops will be assessed as part of a process evaluation and fidelity of the delivery of the intervention will be rated. This data will be used to evaluate the feasibility of training voluntary sector staff to deliver the intervention in conjunction with qualitative interviews with the workshop facilitators. The outcome of this evaluation will be used to inform the training package for workshop facilitators in the main randomised controlled trial.

We anticipate that there will be low loss to follow-up as this is a single session intervention and we will be able to assess the feasibility of a larger study and estimate group means, standard deviations and percentages for key outcomes (to provide some input into the future sample size calculations).[29, 30]

Usual care group

Participants randomised to the usual care arm will receive whatever care is available to them in the community or primary care. We will document the self-reported care received by the participants in the usual care arm at 3 months follow up. There will be no restrictions on the type of care these participants can receive.

Training of facilitators

Voluntary sector providers will be recruited from 'Self-help Services Manchester'. The volunteer facilitators will be trained by WW and AB to deliver the intervention in a group setting. All facilitators will attend a two-day training event held at the University, teaching them the basics of providing group CBT interventions (elements of behavioural activation and cognitive restructuring) and how to deliver the manual. The aim is for them support the manual by guiding the participants in some of the techniques described, not to deliver therapy. We will provide ongoing supervision as all the workshops will be evaluated by the research team as part of a process evaluation.

Outcomes

For peer review only - http://bmjopen.bmj.com/site/about/guidelines.xhtml

For peer review only

Screening questions and demographic information will be recorded for each participant. Data will also be recorded regarding intervention attendance and follow-up.

Quantitative outcomes

To explore acceptability and completion rates quantitative outcomes such as mood, function, participation, costs, and acceptability will be collected.

Baseline questionnaires will include:

- Depression (PHQ 9) a nine item self-report measure[13]
- Health status (EuroQoL) measured on five dimensions on three-point scales[31]
- Client Satisfaction Questionnaire (CSQ-8) an eight-item scale measuring satisfaction on a four-point scale[32]
- Healthcare utilisation measure (CSRI) which records services utilised by patients in primary and secondary care. The measure asks patients to report the number of visits with different healthcare services and practitioners within a three month time-frame[33]

Outcomes will be assessed at three months post randomisation. Quantitative data on recruitment, randomisation, attendance, retention, questionnaire completion rates will be collected.

Statistical analysis

Primary analyses will be descriptive. Recruitment and retention rates will be computed. The feasibility study is not designed to detect differences in clinical outcomes and, as such, will focus on computing appropriate summary statistics (including a measure of variation) for each quantitative outcome. This data, along with estimates of recruitment and attrition rates, will provide the information necessary to inform sample size calculations for a definitive trial.

Qualitative process evaluation

Acceptability of training to facilitators and of the intervention to participants will be explored using qualitative methods. Interviews will be used to explore the acceptability of the intervention, implementation, mechanisms, and context with a sample of the participants and the four facilitators as per MRC guidelines.[34] To achieve this we will conduct up to 15 semi-structured interviews (or until category saturation is achieved) with people receiving the intervention and after the primary outcomes have been assessed at 3 months follow up. We will explore participants' views on being identified as having 'sub-threshold depression' and what this meant to them, the acceptability of the study measures, acceptability of the group format (including content, duration of sessions, venues), relationships with other group members and facilitators; what has changed, if anything, since the intervention. We will interview the workshop facilitators to explore training, their experiences of delivering the intervention, and barriers and facilitators to working with this group of patients. Framework Analysis will be used to analyse the data.[35]

For peer review only - http://bmjopen.bmj.com/site/about/guidelines.xhtml

Discussion

According to the World Health Organisation (WHO), depression is now the most common illness worldwide affecting more than 300,000 people.[7] The impact of depression and the scale of the problem make its treatment and management a key priority for health services. However, recent studies have shown that existing treatments cannot reduce the burden of depression by more than 35%, even under ideal circumstances.[9] Therefore as well as developing new treatments for depression it is essential that focus is placed on depression prevention.[10]

The long-term aim of this study is to adapt and tailor a fairly intensive existing intervention to patients with sub-threshold depression, making it easily accessible, cost effective and sustainable in the community. To achieve these aims we are engaging substantively with members of the public as well as with voluntary sector services and charities to ensure the intervention is both feasible and acceptable. A pilot study will then be used to inform a definitive multi-centre trial to assess efficacy and cost-effectiveness.

The underlying concepts of the intervention have shown efficacy in a number of studies.[20] However, in its original form it requires substantial resources and time commitment from service providers and participants. By adapting the intervention to a workbook and one-day workshop this will reduce costs, make it more accessible (lower burden) to participants, and move the intervention from primary care to voluntary sector organisations. The aim will also be to reduce the likelihood of patients developing diagnosable depression and/or reduce episodes of future depression.

There are several key areas we need to ensure are considered during this pilot study: that the material is easy to understand at a range of reading levels and backgrounds; that the intervention can be delivered by non-NHS, voluntary sector providers; that the training package produced is adequate for the job and gives facilitators the skills they will need; that we can effectively recruit people with sub-threshold depression to a trial; and that the outcome measures are the best measures for assessing a full trial.

Ethics and dissemination

The research was reviewed and received ethical approval from NHS REC NW-Greater Manchester East (ref:17/NW/0604) on 23/11/2017. Participants will be given a detailed participant information sheet (PIS) and will complete and sign a participant consent form prior to participation. Participants will be assigned a unique identification number to ensure anonymity and confidentiality of data collected.

The study already has a website as well as Facebook and Twitter accounts which we are using for recruitment and will also post study updates and results. As well as the protocol, we intend to publish further peer-reviewed articles and present at academic conferences. Engagement events will be held inviting participants, stakeholders and members of the public to summarise the results and get further feedback for the development of a full-scale trial for effectiveness and cost-effectiveness.

For peer review only

For peer review only - http://bmjopen.bmj.com/site/about/guidelines.xhtml

References

1. Waraich P, Goldner EM, Somers JM, et al. Prevalence and incidence studies of mood disorders: a systematic review of the literature. *Can J Psychiatry* 2004; **49**(2):124-138.

2. Saarni SI, Suvisaari J, Sintonen H, et al: Impact of psychiatric disorders on health-related quality of life: general population survey. *Br J Psychiatry* 2007; **190**:326–32.

3. Patel V, Thornicroft G. Packages of care for mental, neurological, and substance use disorders in low- and middle-income countries: PLoS Medicine series. *PLoS Med* 2009; **6**(10):e1000160.

4. Cuijpers P, Vogelzangs N, Twisk J, et al. Comprehensive meta-analysis of excess mortality in depression in the general community versus patients with specific illnesses. *Am J Psychiatry* 2014; **171**:453–62.

5. Berto P, D'Ilario D, Ruffo P, et al: Depression: cost-of-illness studies in the international literature: a review. *J Ment Health Policy Econ* 2000; **3**:3–10.

6. Mathers CD, Loncar D: Projections of global mortality and burden of disease from 2002 to 2030. *PLoS Med* 2006; **3**:e442.

7. World Health Organization. Depression Fact Sheet February 2017. http://www.who.int/mediacentre/factsheets/fs369/en/ (accessed 07/06/2017)

8. Gater R, Tomenson B, Percival C, et al. Persistent depressive disorders and social stress in people of Pakistani origin and white Europeans in UK. *Soc Psych Psychiatr Epidemi* 2009; **44**(3):198-207.

9. Andrews G, Issakidis C, Sanderson K, et. al. Utilising survey data to inform public policy: comparison of the cost-effectiveness of treatment of ten mental disorders. *BJP* 2004; **184**:526–33.

10. Cuijpers P, Beekman AT, Reynolds CF 3rd. Preventing depression: a global priority. *JAMA* 2012; **307**(10):1033-4.

11. Cuijpers P, van Straten A, Smit F, et. al. Preventing the onset of depressive disorders: a meta-analytic review of psychological interventions. *Am J Psychiatry* 2008; **165**(10):1272-80.

12. NICE: The Treatment and Management of Depression in Adults. London: NICE; 2009.

13. Kroenke K, Spitzer RL, Williams JBW. The PHQ-9 - Validity of a brief depression severity measure. J Gen Intern Med 2001; **16**(9):606–13.

14. Baldwin R, Anderson D, Black S, et al. Guideline for the management of late-life depression in primary care. *Int J Geriatr Psychiatry* 2003;**18**(9):829-38.

15. Judd L, Akiskal H, Maser J, et al. A prospective 12-year study of subsyndromal and syndromal depressive symptoms in unipolar major depressive disorders. *Arch Gen Psychiatry* 1998; **55**(8):694-700.

For peer review only - http://bmjopen.bmj.com/site/about/guidelines.xhtml

16. Cuijpers P, Smit F. Subthreshold depression as a risk indicator for major depressive disorder: a systematic review of prospective studies. *Acta Psychiatr Scand* 2004; **109**:325-31.

17. Cuijpers P, De Graaf R, Van Dorsselaer S. Minor depression: risk profiles, functional disability, health care use and risk of developing major depression. *J Affect Disord* 2004; **79**:71-9.

18. Cuijpers P, Smit F, Oostenbrink J, et al. Economic costs of minor depression: A population-based study. *Acta Psychiatr Scand* 2007; **115**:229-36.

19. Cuijpers P, Vogelzangs N, Twisk J, et al. Differential mortality rates in major and subthreshold depression? A meta-analysis of studies that measured both. *Br J Psychiatry* 2013; **202**:22-7.

20. Cuijpers P, Muñoz RF, Clarke GN, et al. Psychoeducational treatment and prevention of depression: the "Coping with Depression" course 30 years later. *Clin Psychol Rev* 2009;**29**(5):449-58.

21. Cuijpers P, van Straten A, Smit F, et al. Preventing the onset of depressive disorders: a meta-analytic review of psychological interventions. *Am J Psychiatry* 2008;**165**(10):1272-80.

22. van Zoonen K, Buntrock C, Ebert DD, et al. Preventing the onset of major depressive disorder: a meta-analytic review of interventions. *Int J Epidemiol* 2014;**43**(2):318-29.

23. White J, Keenan M, Brooks N. Stress control: a controlled comparative investigation of large group therapy for generalized anxiety disorder. *Behav Psychother* 1992; **20**: 97–114.

24. Brown JS, Elliott SA, Boardman J, et al. Meeting the unmet need for depression services with psycho-educational self-confidence workshops: preliminary report. *Br J Psychiatry* 2004;**185**:511-5.

25. Horrell L, Goldsmith K, Tylee A, et al. One-day cognitive–behavioural therapy self-confidence workshops for people with depression: Randomised controlled trial. *Br J Psychiatry* 2014; **204**(3): 222-33.

26. Muñoz, R F (1984). The Depression Prevention Course. Unpublished manuscript, University of California, San Francisco. [Available from the author: University of California, San Francisco/ Department of Psychiatry/San Francisco General Hospital/ 1001 Potrero Avenue, Suite 7M/San Francisco, CA 94110/USA. Email: munoz@itsa.ucsf.edu]

27. Brown JS, Elliott SA, Boardman J, et al. Can the effects of a 1-day CBT psychoeducational workshop on self-confidence be maintained after 2 years? A naturalistic study. *Depress Anxiety* 2008; **25**(7):632-40.

28. Craig P, Dieppe P, Macintyre S, et al. MRC Guidance: Developing and evaluating complex interventions: the new MRC guidance. *BMJ* 2008;**337**:a1655.

29. Lancaster GA, Dodd S, Williamson PR: Design and analysis of pilot studies: recommendations for good practice. *J Eval Clin Pract* 2002; **10**(2):307-312.

30. Shih WJ, Ohman-Strickland PA, Lin Y. Analysis of pilot and early phase studies with small sample sizes. *Statistics in Medicine* 2004;**23**:1827–42.

For peer review only

For peer review only - http://bmjopen.bmj.com/site/about/guidelines.xhtml

31. EuroQol Group: EuroQol–A New Facility for the Measurement of Health-Related Quality of Life, Volume 16. Amsterdam, Netherlands: *Health policy* 1990:199–208.

32. Attkisson CC, Zwick R: The client satisfaction questionnaire. Psychometric properties and correlations with service utilization. *Eval Program Plan* 1982; **5**(3):233–7.

33. Thornicroft G, Brewin C R, Wing J. (Eds.) *Measuring mental health needs.* 1992 London, England: Gaskell/Royal College of Psychiatrists.

34. Moore GF, Audrey S, Barker M, et al. Process evaluation of complex interventions: Medical Research Council guidance. *BMJ* 2015;**350**:h1258.

35. Ritchie J, Spencer L. Qualitative data analysis for applied policy research in A.Bryman and R. G. Burgess [eds.] *Analyzing qualitative data*. 1994.

Author's contributions: WW conceptualised the study. CK drafted the manuscript. All authors reviewed and edited the manuscript. All authors read and approved the final manuscript.

Funding statement: This work was supported by National Institute for Health Research, School for Primary Care Research (Funding Ref 355).

Trial Sponsor: The University of Manchester. FBMHethics@manchester.ac.uk

Competing interests statement: None declared

For peer review only

For peer review only - http://bmjopen.bmj.com/site/about/guidelines.xhtml

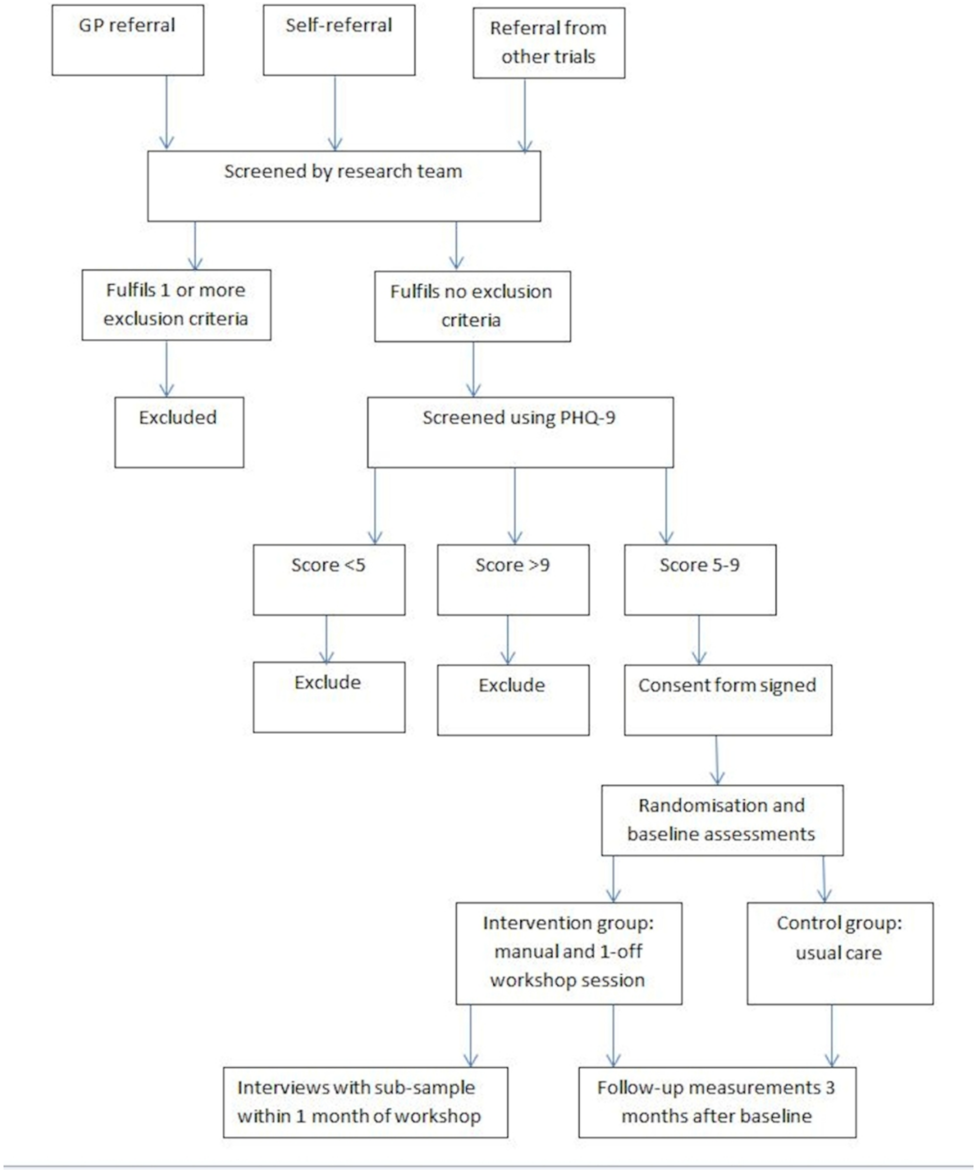

Figure 1- Recruitment and inclusion diagram

112x138mm (300 x 300 DPI)

STANDARD PROTOCOL ITEMS: RECOMMENDATIONS FOR INTERVENTIONAL TRIALS

SPIRIT 2013 Checklist: Recommended items to address in a clinical trial protocol and related documents*

Section/item	Item No	Description	
**Administrative information**			
Title	1	Descriptive title identifying the study design, population, interventions, and, if applicable, trial acronym	P 1
Trial registration	2a	Trial identifier and registry name. If not yet registered, name of intended registry	P 2
	2b	All items from the World Health Organization Trial Registration Data Set	
Protocol version	3	Date and version identifier	P7
Funding	4	Sources and types of financial, material, and other support	P 14
Roles and responsibilities	5a	Names, affiliations, and roles of protocol contributors	P1, P14
	5b	Name and contact information for the trial sponsor	P 14
	5c	Role of study sponsor and funders, if any, in study design; collection, management, analysis, and interpretation of data; writing of the report; and the decision to submit the report for publication, including whether they will have ultimate authority over any of these activities	N/A
	5d	Composition, roles, and responsibilities of the coordinating centre, steering committee, endpoint adjudication committee, data management team, and other individuals or groups overseeing the trial, if applicable (see Item 21a for data monitoring committee)	NA
**Introduction**			
Background and rationale	6a	Description of research question and justification for undertaking the trial, including summary of relevant studies (published and unpublished) examining benefits and harms for each intervention	P4-5
	6b	Explanation for choice of comparators	P 9
Objectives	7	Specific objectives or hypotheses	P 5
Trial design	8	Description of trial design including type of trial (eg, parallel group, crossover, factorial, single group), allocation ratio, and framework (eg, superiority, equivalence, noninferiority, exploratory)	P 7

For peer review only

Methods: Participants, interventions, and outcomes

Study setting	9	Description of study settings (eg, community clinic, academic hospital) and list of countries where data will be collected. Reference to where list of study sites can be obtained	P7
Eligibility criteria	10	Inclusion and exclusion criteria for participants. If applicable, eligibility criteria for study centres and individuals who will perform the interventions (eg, surgeons, psychotherapists)	P 7, Fig.1
Interventions	11a	Interventions for each group with sufficient detail to allow replication, including how and when they will be administered	P9
	11b	Criteria for discontinuing or modifying allocated interventions for a given trial participant (eg, drug dose change in response to harms, participant request, or improving/worsening disease)	NA
	11c	Strategies to improve adherence to intervention protocols, and any procedures for monitoring adherence (eg, drug tablet return, laboratory tests)	P9-10
	11d	Relevant concomitant care and interventions that are permitted or prohibited during the trial	P9
Outcomes	12	Primary, secondary, and other outcomes, including the specific measurement variable (eg, systolic blood pressure), analysis metric (eg, change from baseline, final value, time to event), method of aggregation (eg, median, proportion), and time point for each outcome. Explanation of the clinical relevance of chosen efficacy and harm outcomes is strongly recommended	P10
Participant timeline	13	Time schedule of enrolment, interventions (including any run-ins and washouts), assessments, and visits for participants. A schematic diagram is highly recommended (see Figure)	P10, Fig.1
Sample size	14	Estimated number of participants needed to achieve study objectives and how it was determined, including clinical and statistical assumptions supporting any sample size calculations	P7, 10
Recruitment	15	Strategies for achieving adequate participant enrolment to reach target sample size	P7-8

Methods: Assignment of interventions (for controlled trials)

Allocation:

Sequence generation	16a	Method of generating the allocation sequence (eg, computer-generated random numbers), and list of any factors for stratification. To reduce predictability of a random sequence, details of any planned restriction (eg, blocking) should be provided in a separate document that is unavailable to those who enrol participants or assign interventions	P 8

For peer review only

Allocation concealment mechanism	16b	Mechanism of implementing the allocation sequence (eg, central telephone; sequentially numbered, opaque, sealed envelopes), describing any steps to conceal the sequence until interventions are assigned	P 8
Implementation	16c	Who will generate the allocation sequence, who will enrol participants, and who will assign participants to interventions	P 8
Blinding (masking)	17a	Who will be blinded after assignment to interventions (eg, trial participants, care providers, outcome assessors, data analysts), and how	P 8
	17b	If blinded, circumstances under which unblinding is permissible, and procedure for revealing a participant's allocated intervention during the trial	NA
**Methods: Data collection, management, and analysis**			
Data collection methods	18a	Plans for assessment and collection of outcome, baseline, and other trial data, including any related processes to promote data quality (eg, duplicate measurements, training of assessors) and a description of study instruments (eg, questionnaires, laboratory tests) along with their reliability and validity, if known. Reference to where data collection forms can be found, if not in the protocol	P10
	18b	Plans to promote participant retention and complete follow-up, including list of any outcome data to be collected for participants who discontinue or deviate from intervention protocols	P 10
Data management	19	Plans for data entry, coding, security, and storage, including any related processes to promote data quality (eg, double data entry; range checks for data values). Reference to where details of data management procedures can be found, if not in the protocol	P8, 10
Statistical methods	20a	Statistical methods for analysing primary and secondary outcomes. Reference to where other details of the statistical analysis plan can be found, if not in the protocol	P10
	20b	Methods for any additional analyses (eg, subgroup and adjusted analyses)	NA
	20c	Definition of analysis population relating to protocol non-adherence (eg, as randomised analysis), and any statistical methods to handle missing data (eg, multiple imputation)	NA
**Methods: Monitoring**			
Data monitoring	21a	Composition of data monitoring committee (DMC); summary of its role and reporting structure; statement of whether it is independent from the sponsor and competing interests; and reference to where further details about its charter can be found, if not in the protocol. Alternatively, an explanation of why a DMC is not needed	NA

For peer review only

	21b	Description of any interim analyses and stopping guidelines, including who will have access to these interim results and make the final decision to terminate the trial	NA
Harms	22	Plans for collecting, assessing, reporting, and managing solicited and spontaneously reported adverse events and other unintended effects of trial interventions or trial conduct	NA
Auditing	23	Frequency and procedures for auditing trial conduct, if any, and whether the process will be independent from investigators and the sponsor	NA
**Ethics and dissemination**			
Research ethics approval	24	Plans for seeking research ethics committee/institutional review board (REC/IRB) approval	P 2,11
Protocol amendments	25	Plans for communicating important protocol modifications (eg, changes to eligibility criteria, outcomes, analyses) to relevant parties (eg, investigators, REC/IRBs, trial participants, trial registries, journals, regulators)	
Consent or assent	26a	Who will obtain informed consent or assent from potential trial participants or authorised surrogates, and how (see Item 32)	P 8
	26b	Additional consent provisions for collection and use of participant data and biological specimens in ancillary studies, if applicable	NA
Confidentiality	27	How personal information about potential and enrolled participants will be collected, shared, and maintained in order to protect confidentiality before, during, and after the trial	P 8
Declaration of interests	28	Financial and other competing interests for principal investigators for the overall trial and each study site	P 14
Access to data	29	Statement of who will have access to the final trial dataset, and disclosure of contractual agreements that limit such access for investigators	P8
Ancillary and post-trial care	30	Provisions, if any, for ancillary and post-trial care, and for compensation to those who suffer harm from trial participation	NA
Dissemination policy	31a	Plans for investigators and sponsor to communicate trial results to participants, healthcare professionals, the public, and other relevant groups (eg, via publication, reporting in results databases, or other data sharing arrangements), including any publication restrictions	P2, 11
	31b	Authorship eligibility guidelines and any intended use of professional writers	NA
	31c	Plans, if any, for granting public access to the full protocol, participant-level dataset, and statistical code	NA

For peer review only

Appendices

Informed consent materials	32	Model consent form and other related documentation given to participants and authorised surrogates	NA
Biological specimens	33	Plans for collection, laboratory evaluation, and storage of biological specimens for genetic or molecular analysis in the current trial and for future use in ancillary studies, if applicable	NA

*It is strongly recommended that this checklist be read in conjunction with the SPIRIT 2013 Explanation & Elaboration for important clarification on the items. Amendments to the protocol should be tracked and dated. The SPIRIT checklist is copyrighted by the SPIRIT Group under the Creative Commons "Attribution-NonCommercial-NoDerivs 3.0 Unported" license.

For peer review only

BMJ Open

Preventing depression in the community by voluntary sector providers (PERSUADE): intervention development and protocol for a parallel randomized controlled feasibility trial.

Journal:	*BMJ Open*
Manuscript ID	bmjopen-2018-023791.R1
Article Type:	Protocol
Date Submitted by the Author:	29-Oct-2018
Complete List of Authors:	Kenning, Cassandra; University of Manchester, Division of Population Health, Health Services Research and Primary Care<br>Blakemore, Amy; University of Manchester,<br>Bower, Peter; University of Manchester, Division of Population Health, Health Services Research and Primary Care<br>Safari, Melina; University of Manchester, Division of Population Health, Health Services Research and Primary Care<br>Cuijpers, Pim; Vrije Universiteit Amsterdam<br>Brown, June S. L.; Kings College London<br>Hann , Mark ; University of Manchester, Centre for Biostatistics and Manchester Academic Health Science Centre<br>Lidbetter, Nicky; Self Help Services<br>Muñoz, Ricardo; Palo Alto University, Institute for International Internet Interventions for Health<br>Waheed, Waquas; University of Manchester, Division of Population Health, Health Services Research and Primary Care
**Primary Subject Heading**:	Public health
Secondary Subject Heading:	Qualitative research, Mental health
Keywords:	Depression & mood disorders < PSYCHIATRY, PUBLIC HEALTH, PREVENTIVE MEDICINE, MENTAL HEALTH

SCHOLARONE™
Manuscripts

Preventing depression in the community by voluntary sector providers (PERSUADE): intervention development and protocol for a parallel randomized controlled feasibility trial.

Cassandra Kenning. Division of Population Health, Health Services Research & Primary Care. The University of Manchester, Manchester UK.

Amy Blakemore. Division of Nursing, Social Work & Midwifery. The University of Manchester, Manchester UK.

Peter Bower. Division of Population Health, Health Services Research & Primary Care. The University of Manchester, Manchester UK.

Melina Safari. Division of Population Health, Health Services Research & Primary Care. The University of Manchester, Manchester UK.

Pim Cuijpers, Faculty of Behaviour and Movement Sciences. Vrije Universiteit, Amsterdam, The Netherlands.

June Brown, Department of Psychology. King's College London, London UK.

Mark Hann. Centre for Biostatistics and Manchester Academic Health Science Centre. The University of Manchester, Manchester UK.

Nicky Lidbetter, Self Help Services, Manchester UK.

Ricardo Muñoz, Clinical Psychology. Palo Alto University. Palo Alto CA, US.

Waquas Waheed* Division of Population Health, Health Services Research & Primary Care. The University of Manchester, Manchester UK.

*corresponding author

Dr Waquas Waheed

The University of Manchester

5th Floor Williamson Building

Oxford Road

Manchester

M13 9PL

Waquas.waheed@manchester.ac.uk

For peer review only - http://bmjopen.bmj.com/site/about/guidelines.xhtml

For peer review only

Tel: 0161 306 7949

Word count: 4028

Abstract

Introduction

Depression is now the most common illness worldwide affecting more than 300 million people. Studies modelling the impact of depression interventions have shown that the burden of depression cannot be minimised by more than 35% with existing treatments. There is a need to develop better preventative interventions. The overall aim of this programme of work is to develop interventions to reduce depression symptom scores and prevent development of depression in people with sub-threshold depression. The objectives are to adapt a low intensity community based depression prevention intervention, establish the acceptability and uptake of this model, and to conduct a parallel randomized controlled feasibility trial to inform a full-scale trial.

Methods and analysis

Focus groups will be run with members of the public, voluntary sector providers, and researchers to inform the adaptation of an existing depression prevention course. Sixty four people with sub-threshold depression, as represented by a score of between 5 and 9 on the PHQ-9 depression measure, will be recruited to take part in the feasibility trial. Participants will be randomised equally to the intervention or usual care control groups. Participants in the intervention group will receive the new revised manual and attend a one-day workshop delivered by voluntary sector service providers. Outcome measures will be completed three months after baseline. Quantitative data on recruitment, randomisation, attendance, retention, questionnaire completion rates will be collected. Primary analyses will be descriptive and a process evaluation conducted to assess the processes involved in implementing the intervention. Interviews will be conducted to explore acceptability and framework analysis will be used to analyse the data.

Ethics and dissemination

The study has been reviewed and approved by NHS Research and Ethics Committee: NW-Greater Manchester East. The results will be actively disseminated through peer-reviewed journals, conference presentations, social media, the internet and community engagement activities.

Trial registration: ISRCTN23278208

Key words: sub-threshold, depression, prevention, protocol, feasibility, .

For peer review only - http://bmjopen.bmj.com/site/about/guidelines.xhtml

For peer review only

Strengths and limitations of this study

- Adapting the intervention to a workbook and one-day workshop will reduce costs, make it more accessible (lower burden) to participants, and move the intervention from primary care to voluntary sector organisations.
- Recruiting people with sub-threshold depression can be challenging and so the study will evaluate a number of different recruitment strategies and assess them to see how much time each takes and the associated yield.
- We are engaging substantively with members of the public as well as with voluntary sector services and charities to ensure the intervention is both feasible and acceptable.
- This is a feasibility trial and is not powered to show effectiveness of the intervention.

For peer review only

For peer review only - http://bmjopen.bmj.com/site/about/guidelines.xhtml

Introduction

Depression has high prevalence and incidence,[1] affects quality of life,[2] productivity, fulfilment of social roles, and increases mortality,[3, 4] leading to high service use and economic costs.[5] Depression is a major contributor to global disease burden, predicted to become the leading contributor in high-income countries by 2030.[6] Recent reports by the World Health Organisation (WHO), show that depression is the leading cause of disability worldwide, affecting more than 300 million people.[7] In a recent community cohort study of whites and British Pakistanis, prevalence of depression was 12.6%. At 6 months follow up 60.5% cases were persistently depressed and incidence of new cases was 17%.[8]

Modelling studies have shown that existing treatments cannot reduce the burden of depression by more than 35%, even under ideal circumstances.[9] Major depression can be prevented with current knowledge. [10,11] Prevention may offer an opportunity to reduce the disease burden of depression further.[12] A number of studies have examined the capability of prevention programmes to successfully reduce the incidence of depression.[13,14] This is important from a public health perspective as duration of the disorder is inversely related to outcome, so that by the time cases are recognised, they are harder to treat. Developing an intervention aimed at both prevention and early treatment could potentially reduce the overall burden of depression.[6]

NICE guidelines (CG 90 and 91) advocate case-finding by GP for depression in high risk groups with physical comorbidities, and those with past history of depression. They recommend offering anti-depressants or psychological interventions to people with moderate to severe depression.[15] In the UK access to psychological therapies for depression is delievered by IAPT teams (Improved Access to Psychological Therapies). According to the service guidelines they offer screening and then access to services for people scoring above 10 on the Patient Health Questionnaire (PHQ-9) screening tool. [16] A range of evidence-based psychological interventions are delivered according to a stepped-care model for those with mild-moderate and moderate-severe depression.[17]

Less attention has been paid to sub-threshold depression, defined as those who respond positively to case-finding questions on the PHQ-9 but do not have sufficient symptom scores on diagnostic criteria to screen positive for major depressive episode.[18] Sub-threshold depression is a major risk factor for progression and development of more severe depression[19,20] and has been shown to have similar consequences.[21-23]

Previous UK trials have generally included cases with above threshold depression and have not looked exclusively at people with sub-threshold depression. We could identify only two UK RCTs in the two published meta-analyses of 25 depression prevention trials.[24, 25] Both UK trials were aimed exclusively at pregnant women and published in 2000. An update of this meta-analysis included 34 RCTs with no further RCTs from UK.[26]

An intervention for sub-threshold depression needs to reduce the incidence of new onset depression but also improve outcome for those who do develop depression by reducing the length of the episode. GPs coordinating with voluntary sector organisations to deliver the intervention will potentially increase

For peer review only - http://bmjopen.bmj.com/site/about/guidelines.xhtml

access for hard to reach groups.[27-29] Depression treatment studies using lay health workers to deliver cognitive behavioural interventions have already been found effective in settings with few professional mental health providers. [30-32] However, voluntary sector workers will need to be adequately trained and supervised in delivering a patient-centred intervention according to a treatment manual. Substance abuse treatment counsellors have successfully administered a version of the depression prevention intervention to treat depression in individuals in substance abuse treatment.[33]

We need to understand user and provider views about prevention strategies in subthreshold depression and their applicability to UK health systems. There is uncertainty around the content, format, duration and use of voluntary sector workers to deliver such an intervention. We will aim to address these issues through involving users, providers, primary care staff and experts in this field and incorporating their opinions into the intervention and trial design.

The model we propose to use will be a psycho-educational intervention for prevention of depression in the form of a community based workshop to be delivered to people with sub-threshold depression. The proposed intervention will be adapted from the “Depression Prevention Course” and include evidence based CBT techniques, previously developed by Muñoz for preventing depression. The intervention is fully described in the book: The Prevention of Depression: Research and Practice [34] and an online version of the manual is freely available. [35] Many trials have already used versions of the manals from the depression prevention course and have shown efficacy in diverse populations. [36-38] The adapted intervention will be delivered via an accessible community workshop format as developed for depressed people by Brown (Self-confidence workshops).[28,29,39]

Research objectives

The eventual aim of our research programme is to reduce depression symptom scores and prevent development of depression in people with sub-threshold depression defined as PHQ score 5-9.[16] This feasibility trial will follow the MRC framework [40] for the development and evaluation of complex interventions, and has the following objectives:

1. To adapt a low intensity community based depression prevention intervention based on CBT model for adults with sub-threshold depression deliverable by voluntary sector organisations.

2. To establish the acceptability and uptake of this model by adults with sub-threshold depression.

3. To test the feasibility of conducting a successful trial of community based depression prevention intervention for adults with sub-threshold depression.

Methods and analysis

Patient and Public Involvement

For peer review only - http://bmjopen.bmj.com/site/about/guidelines.xhtml

Over a number of years we have conducted research in collaboration with community based voluntary organisations (Roby Church, Mind, Pakistani resource centre, Scaitcliffe Community Centre) in the North West. During past stakeholder events it had been discussed that interventions should be made available that prevent depression in vulnerable people.

During the preparation of the funding application we worked with voluntary sector organisations and providers (YARAN Community services, Self Help Services, Manchester) to inform the proposed intervention content, delivery and research design. We also made presentations to a lay public group and incorporated their suggestions into the proposal. YARAN Community services and Self Help Services, Manchester will continue to offer advice throughout the project. Phase 1 of the study involves community focus groups and at the end of the study community events will be used to feed back the results and to develop the proposal for a full trial for effectiveness and cost-effectiveness. Members of the public will be involved in the recruitment to the study by providing feedback on recruitment materials during the focus groups but will not be involved in the conduct of the study.

Qualitative interviews with participants in the intervention group will focus on their experiences of a, taking part in a study and b, all aspects of the intervention (manual and workshop) to explore acceptability, burden of the intervention and to further modify or adapt the intervention and trial design for a full-trial.

Phase 1- intervention development

Intervention and manual development: The 'Depression Prevention Course' developed by Muñoz[34,35] will be adapted for this study. Box 1 below shows key components of the original Depression Prevention Course.

Box 1- Depression Prevention Course

Originally based on Social Learning Theory

The intervention was developed using materials from the book 'Control your Depression' 1978

Combines three methods used to treat depression:

- Increasing pleasant activities
- Social skills training
- Cognitive approaches

Delivered by trained CBT therapist

8 group sessions delivered over 8 weeks

51 page 'Outlines for participants' manual (text only)

For peer review only - http://bmjopen.bmj.com/site/about/guidelines.xhtml

Focus groups: To tailor the intervention, focus groups will be held with users and providers.

A series of focus groups (3-5) will be run with a variety of groups including the general population, voluntary service providers from Self-help Manchester, and academic participants. The aim of the groups will be to get feedback on intervention content, delivery options, manual design, as well as recruitment methods. This will assist in the intervention adaptation and delivery, and also in refining the recruitment methods and materials. As the different aspects are discussed the groups will be asked to try to reach a consensus on their feedback, for example when offered a range of images, which do they think are the most engaging or in the case of terminology do they prefer ‘depression’ or ‘low mood’ or ‘feeling down’ among other suggestions. The groups will be held at a location convenient to participants and last around 2 hours. Sessions will be audio recorded, with consent, and transcribed.

Prevent depression Intervention:

Once we have run the focus groups, and discussed the outcomes within the trial team, the original Muñoz manual will be redesigned as an accessible and easy to understand manual based intervention. To support the manual and guide people in its use, participants will attend a one day (or two half-day) workshop. The aim is that offering a one-off psychoeducational group will improve access for a larger group of people. Asking participants to commit to 8 weeks of intervention when they do not yet have a diagnosable condition requires substantial resources and time commitment from service providers and participants. This workshop approach has been effective in a number of studies.[27,28]

Phase 2- Feasibility trial

PERSUADE Protocol v2 (20/08/2017)

Design: A mixed methodsfeasibility trial and process evaluation to explore feasibility and acceptability of a depression prevention intervention.

Setting: Primary care and community, Greater Manchester UK.

Feasibility parameters: best recruitment methods to identify people with sub-threshold depression, participants willingness to be randomised, retention rates and outcome measure completion rates, acceptability of the intervention to both participants and facilitators, training of facilitators from voluntary sector services to deliver the intervention.

Inclusion criteria: Patients from primary care and the general community who are aged above 18 years and who score between 5 and 9 on the PHQ 9.[16]

Exclusion criteria:

For peer review only - http://bmjopen.bmj.com/site/about/guidelines.xhtml

For peer review only

Candidates will be asked if they have any diagnoses for:

- Depression (score higher than 9 on PHQ-9, or formal diagnosis of depression)
- Psychosis (self-report)
- Drug or alcohol use as primary diagnosis (self-report)
- Suicidal ideations (self-report)
- Non-English language speakers

Participant recruitment: As this is a feasibilitytrial, a formal sample size calculation was not undertaken. The aim is to recruit a total of 64 patients to be randomised with equal probability to the intervention or usual care (i.e. 32 to each). This is broadly in line with current guidance on feasibility trials [41] and was considered sufficient to allow the formation of multiple groups for testing of the intervention, and to provide sufficiently robust estimates of recruitment timelines and rates of loss to follow up, within the limits of the available funding.

Considering the challenge of recruiting persons with sub-threshold depression, a number of different recruitment strategies will be tried and assessed to see how much time each takes and the associated yield.

Recruitment through GP practices:

- GPs to refer patients recently screened with the PHQ-9 who scored between 5 and 9. They will either give out the study information during the consultation or post the patient materials to them.
- Searches will be carried out on GP practice databases to see if it is feasible to identify patients who have concerns about their mood but who have not been referred on to IAPT (Improving Access to Psychological Therapies) services due to scores below 9 on the PHQ-9. Searches will be done by practice staff or CRN support staff to identify potential participants and mail out the study information.
- Research staff to offer study information and to screen patients with PHQ-9 in GP waiting rooms if they are interested in taking part.

Network support will be sought from Greater Manchester NIHR Clinical Research Network (CRN) to ensure that the research project is conducted within the proposed timeframe and recruitment targets are achieved. CRN will help with engaging primary care and liaising with GP at site.

Recruitment through advertising:

- Voluntary sector organisations (Scaitcliffe centre, YARAAN community, Self-help Services, Manchester) to direct self-referring patients by providing information about the study.
- Advertise in GP practice, library, community centres, citizens' advice bureau, Sure Start and places of worship.
- Advertise on dedicated Website, Facebook and Twitter

For peer review only - http://bmjopen.bmj.com/site/about/guidelines.xhtml

- Advertise the study in local newspapers/radio, and on Eventbrite.

Recruitment through other trials:

- Invite participants who are screened out from any ongoing depression trials run under Clinical Research Network. For those trials that agree, once patients have been screened as ineligible from other depression trials due to a low score on PHQ-9, they will send the details of PERSUADE trial to ineligible patients.

Consent

Patients consenting to be contacted by the research team will be contacted over the phone or if needed, visited at home or a convenient location. They will be given a participant information sheet and have the study clearly explained to them, and will be given the opportunity to ask questions. The researcher will screen the participant for eligibility (as above) and if patients agree and meet the inclusion criteria they will be asked to sign and return the 'participant consent form' to enter the trial. Baseline assessments will then be self-completed or if assistance is needed, by telephone.

Confidentiality and data storage

All participants will be assigned a unique identification number which will be used for randomisation, data collection documents and analysis. Consent forms will be stored separately from participant data in locked filing cabinets. Publication of results will retain identification numbers to preserve anonymity, particulary where direct quotes are used. Only the primary research team will have access to the final trial data set.

Ineligible and non-recruited participants

In cases where either moderate to severe depression (scoring 10 or more on PHQ-9) or thoughts of current self-harm or suicide are expressed, potential participants will be referred back to their GP. The PHQ-9 includes the statement: "Thoughts that you would be better off dead or of hurting yourself in some way". If candidates score highly on this question (2 or 3) they will be advised to see there GP as soon as possible to discuss their mood.

Randomisation Participants will be randomised with equal probability to the CBT-based workshops or usual care (i.e. 32 to each). Computer generated block randomisation will be used. The allocation sequence will be set up by someone independent of the research team. Once a participant is randomised the trial research associate will be informed by email and they will then contact participants. Participants will be informed of the allocation by letter or telephone. As this is an active intervention they will not be blinded. Also as this is a feasibility trial and not a trial of efficacy the research associate/PhD student will not be blind to allocation.

For peer review only - http://bmjopen.bmj.com/site/about/guidelines.xhtml

For peer review only

The figure below describes the process for the entry of participants into the trial and data collection points.

Figure 1 Recruitment and inclusion diagram * Insert here*

We will follow the predetermined Stop-Go criteria for definitive RCTs:

i. GO if 52 or more patients are recruited into the study (the target 64 minus 10%)
ii. STOP if <39 (60%) patients recruited into study
iii. STOP Intervention is unacceptable to participants

Intervention

Format and content of the intervention

We will train voluntary sector workers to facilitate community workshops that will last up to 8 hours and will be delivered either on a single day or be split over 2 days (evenings and weekends). Participants will attend one of these workshops. There will be no restrictions on the type of care participants can receive from other services.

Data on content, design and format of prevention interventions has already been extracted and will be published as a review, this will guide us in adapting the "Depression Prevention Course"[34] for this study. Motivational techniques will be incorporated in the beginning of the workshop to help engage participants, based on previous research and our own experience of conducting social groups. Group exercises, role-play demonstrations and discussions of case vignettes will be participatory to help maintain participant's interest and motivation throughout the workshop. Participants will receive a professionally designed manual, in colour and with graphics, that describes the workshop's program and all content. Workshops will be assessed as part of a process evaluation and fidelity of the delivery of the intervention will be rated. Using a template, the workshops will be assessed for adherence to the intervention. A record will also be made of the amount of engagement and participation in the groups. Length of the workshops and if participants remain for the entire session will also be recorded and reported. These data will be used to evaluate the feasibility of training voluntary sector staff to deliver the intervention in conjunction with qualitative interviews with the workshop facilitators. The outcome of this evaluation will be used to inform the training package for workshop facilitators in the main randomised controlled trial.

We anticipate that there will be low loss to follow-up as this is a single session intervention and we will be able to assess the feasibility of a larger study and estimate group means, standard deviations and percentages for key outcomes (to provide some input into the future sample size calculations).[41,42]

Usual care group

Participants randomised to the usual care arm will receive whatever care is available to them in the community or primary care. We will document the self-reported care received by the participants in the

For peer review only - http://bmjopen.bmj.com/site/about/guidelines.xhtml

usual care arm at 3 months follow up. There will be no restrictions on the type of care these participants can receive.

Training of facilitators

Voluntary sector facilitators will be recruited from ‘Self-help Services Manchester’. There are no exclusion criteria but facilitators need: Experience of working or volunteering in a community setting; Experience of working with groups; Knowledge of mental health problems and an interest in the area of mental health. We aim to recruit four facilitators to work in pairs to deliver the workshops. The volunteer facilitators will be trained by WW and AB to deliver the intervention in a group setting. All facilitators will attend a two-day training event held at the University, teaching them the basics of providing group CBT interventions (elements of behavioural activation and cognitive restructuring) and how to deliver the manual. The aim is for them to support the manual by guiding the participants in some of the techniques described, not to deliver therapy. We will provide ongoing supervision as all the workshops will be evaluated by the research team as part of a process evaluation.

Outcomes

The primary outcome for this feasibility trial will be recruitment rates. Screening questions,demographic information, baseline and follow-up assessments will be recorded for each participant. Data will also be recorded regarding intervention attendance and retention rates for the three month follow-up.

Quantitative outcomes

To explore outcomes and completion rates, quantitative measures including mood, function, participation, costs, and acceptability will be collected.

Baseline questionnaires will include:

- Depression (PHQ 9) a nine item self-report measure[16] PHQ-9 scores of 5, 10, 15, and 20 represent mild, moderate, moderately severe, and severe depression. A widely used screening measure for depression, it has displayed good internal reliability (Cronbach's α 0.89).
- Health status (EuroQoL) EQ-5D-3L, measured on five dimensions on three-point scales. Is a widely established measure showing high levels of internal validity and reliability in diverse populations and in many languages. [43]
- Client Service Receipt Inventory (CSRI), is an adaptable healthcare utilisation measure which records services used by patients in the community, primary and secondary care. The measure asks patients to report the number of visits with different healthcare services and practitioners within a three month time-frame .[44]

Outcomes will be assessed at three months post randomisation,using the same baseline measures with the addition of Client Satisfaction Questionnaire (CSQ-8) an eight-item scale measuring satisfaction on a

For peer review only - http://bmjopen.bmj.com/site/about/guidelines.xhtml

four-point scale. The measure reports excellent internal reliability (coefficient α .93).[45] All participants will be asked to complete all outcome measures. The researcher doing the analysis will be blind to allocation and so all measures must be given to all participants. Quantitative data on recruitment, randomisation, attendance, retention, questionnaire completion rates will be collected.

Statistical analysis

Primary analyses will be descriptive. Recruitment and retention rates will be computed. The trial is not designed to detect differences in clinical outcomes and, as such, will focus on computing appropriate summary statistics (including a measure of variation) for each quantitative outcome. We will report on preliminary incidence figures in both groups by reporting if any participants screen positive for major depression at follow-up. These data, along with estimates of recruitment and attrition rates, will provide the information necessary to inform sample size calculations for a definitive trial.

Qualitative process evaluation

Acceptability of training to facilitators and of the intervention to participants will be explored using qualitative methods. Interviews will be used to explore the acceptability of the intervention, implementation, mechanisms, and context with a sample of the participants and the four facilitators as per MRC guidelines.[46] To achieve this we will conduct up to 15 semi-structured interviews (or until category saturation is achieved) with people receiving the intervention and after the primary outcomes have been assessed at 3 months follow up. We will explore participants' views on being identified as having 'sub-threshold depression' and what this meant to them, the acceptability of the study measures, acceptability of the group format (including content, duration of sessions, venues), relationships with other group members and facilitators; what has changed, if anything, since the intervention. We will interview the workshop facilitators to explore training, their experiences of delivering the intervention, and barriers and facilitators to working with this group of patients. Framework Analysis will be used to analyse the data.[47]

Analysis will be conducted according to the constant comparative method [48], whereby analysis is carried out concurrently with data collection so that emerging issues can be iteratively explored. Transcripts will be analysed independently and coded using qualitative data analysis software. Themes will be discussed by the research team to reach consensus and a coding framework that includes higher level themes and relevant data will be assembled. Each transcript will analysed individually and then in groups, with the facilitator transcripts analysed separately from the participant transcripts but with comparisons being made across datasets.

Discussion

According to the World Health Organisation (WHO), depression is now the most common illness worldwide affecting more than 300 million people.[7] The impact of depression and the scale of the problem make its treatment and management a key priority for health services. However, recent studies have shown that existing treatments cannot reduce the burden of depression by more than 35%, even

For peer review only - http://bmjopen.bmj.com/site/about/guidelines.xhtml

under ideal circumstances.[9] Therefore as well as developing new treatments for depression it is essential that focus is placed on depression prevention.[12]

The long-term aim of this study is to adapt and tailor a fairly intensive existing intervention to patients with sub-threshold depression, making it easily accessible, cost effective and sustainable in the community. To achieve these aims we are engaging substantively with members of the public as well as with voluntary sector services and charities to ensure the intervention is both feasible and acceptable. A pilot study will then be used to inform a definitive multi-centre trial to assess efficacy and cost-effectiveness.

The underlying concepts of the intervention have shown efficacy in a number of studies.[24,36,37,38] However, in its original form it requires substantial resources and time commitment from service providers and participants. By adapting the intervention to a workbook and one-day workshop this will reduce costs, make it more accessible (lower burden) to participants, and move the intervention from primary care to voluntary sector organisations. The aim will also be to reduce the likelihood of patients developing diagnosable depression and/or reduce episodes of future depression.

There are several key areas we need to ensure are considered during this feasibility trial: that the material is easy to understand at a range of reading levels and backgrounds; that the intervention can be delivered by non-NHS, voluntary sector providers; that the training package produced is adequate for the job and gives facilitators the skills they will need; that we can effectively recruit people with sub-threshold depression to a trial; and that the outcome measures are the best measures for assessing a full trial.

Ethics and dissemination

The research was reviewed and received ethical approval from NHS REC NW-Greater Manchester East (ref:17/NW/0604) on 23/11/2017. Participants will be given a detailed participant information sheet (PIS) and will complete and sign a participant consent form prior to participation. Participants will be assigned a unique identification number to ensure anonymity and confidentiality of data collected.

The study already has a website as well as Facebook and Twitter accounts which we are using for recruitment and will also post study updates and results. As well as the protocol, we intend to publish further peer-reviewed articles and present at academic conferences. Engagement events will be held inviting participants, stakeholders and members of the public to summarise the results and get further feedback for the development of a full-scale trial for effectiveness and cost-effectiveness.

For peer review only - http://bmjopen.bmj.com/site/about/guidelines.xhtml

References

1. Waraich P, Goldner EM, Somers JM, et al. Prevalence and incidence studies of mood disorders: a systematic review of the literature. *Can J Psychiatry* 2004; **49**(2):124-138.

2. Saarni SI, Suvisaari J, Sintonen H, et al: Impact of psychiatric disorders on health-related quality of life: general population survey. *Br J Psychiatry* 2007; **190**:326–32.

3. Patel V, Thornicroft G. Packages of care for mental, neurological, and substance use disorders in low- and middle-income countries: PLoS Medicine series. *PLoS Med* 2009; **6**(10):e1000160.

4. Cuijpers P, Vogelzangs N, Twisk J, et al. Comprehensive meta-analysis of excess mortality in depression in the general community versus patients with specific illnesses. *Am J Psychiatry* 2014; **171**:453–62.

5. Berto P, D'Ilario D, Ruffo P, et al: Depression: cost-of-illness studies in the international literature: a review. *J Ment Health Policy Econ* 2000; **3**:3–10.

6. Mathers CD, Loncar D: Projections of global mortality and burden of disease from 2002 to 2030. *PLoS Med* 2006; **3**:e442.

7. World Health Organization. Depression Fact Sheet 22 March 2018. http://www.who.int/news-room/fact-sheets/detail/depression (accessed 24/08/2018)

8. Gater R, Tomenson B, Percival C, et al. Persistent depressive disorders and social stress in people of Pakistani origin and white Europeans in UK. *Soc Psych Psychiatr Epidemi* 2009; **44**(3):198-207.

9. Andrews G, Issakidis C, Sanderson K, et. al. Utilising survey data to inform public policy: comparison of the cost-effectiveness of treatment of ten mental disorders. *BJP* 2004; **184**:526–33.

10. Muñoz RF, Beardslee WR, & Leykin Y. Major depression can be prevented. *American Psychologist* 2012; **67:**285-95.

11. Muñoz, R.F., Schueller, S.M., Barrera, A.Z., Le, H.N., & Torres, L. (2014). Major depression can be prevented: Implications for research and practice. In I.H. Gotlib & C.L. Hammen (Eds.), *Handbook of depression*-Third edition (pp. 471-491). New York: The Guilford Press.

12. Cuijpers P, Beekman AT, Reynolds CF 3rd. Preventing depression: a global priority. *JAMA* 2012; **307**(10):1033-4.

13. Cuijpers P, van Straten A, Smit F, et. al. Preventing the onset of depressive disorders: a meta-analytic review of psychological interventions. *Am J Psychiatry* 2008; **165**(10):1272-80.

14. Muñoz, R. F., Cuijpers, P., Smit, F., Barrera, A.Z., & Leykin, Y. (2010). Prevention of major depression. Annual Review of Clinical Psychology, 6, 181-212.

15. NICE: The Treatment and Management of Depression in Adults. London: NICE; 2009.

For peer review only - http://bmjopen.bmj.com/site/about/guidelines.xhtml

16. Kroenke K, Spitzer RL, Williams JBW. The PHQ-9 - Validity of a brief depression severity measure. J Gen Intern Med 2001; 16(9):606–13.

17. National Collaborating Centre for Mental Health. The Improving Access to Psychological Therapies Manual. V1, June 2018. NHS England.

18. Baldwin R, Anderson D, Black S, et al. Guideline for the management of late-life depression in primary care. *Int J Geriatr Psychiatry* 2003;**18**(9):829-38.

19. Judd L, Akiskal H, Maser J, et al. A prospective 12-year study of subsyndromal and syndromal depressive symptoms in unipolar major depressive disorders. *Arch Gen Psychiatry* 1998; **55**(8):694-700.

20. Cuijpers P, Smit F. Subthreshold depression as a risk indicator for major depressive disorder: a systematic review of prospective studies. *Acta Psychiatr Scand* 2004; **109**:325-31.

21. Cuijpers P, De Graaf R, Van Dorsselaer S. Minor depression: risk profiles, functional disability, health care use and risk of developing major depression. *J Affect Disord* 2004; **79**:71-9.

22. Cuijpers P, Smit F, Oostenbrink J, et al. Economic costs of minor depression: A population-based study. *Acta Psychiatr Scand* 2007; **115**:229-36.

23. Cuijpers P, Vogelzangs N, Twisk J, et al. Differential mortality rates in major and subthreshold depression? A meta-analysis of studies that measured both. *Br J Psychiatry* 2013; **202**:22-7.

24. Cuijpers P, Muñoz RF, Clarke GN, et al. Psychoeducational treatment and prevention of depression: the "Coping with Depression" course 30 years later. *Clin Psychol Rev* 2009;**29**(5):449-58.

25. Cuijpers P, van Straten A, Smit F, et al. Preventing the onset of depressive disorders: a meta-analytic review of psychological interventions. *Am J Psychiatry* 2008;**165**(10):1272-80.

26. van Zoonen K, Buntrock C, Ebert DD, et al. Preventing the onset of major depressive disorder: a meta-analytic review of interventions. *Int J Epidemiol* 2014;**43**(2):318-29.

27. White J, Keenan M, Brooks N. Stress control: a controlled comparative investigation of large group therapy for generalized anxiety disorder. *Behav Psychother* 1992; **20**: 97–114.

28. Brown JS, Elliott SA, Boardman J, et al. Meeting the unmet need for depression services with psycho-educational self-confidence workshops: preliminary report. *Br J Psychiatry* 2004;**185**:511-5.

29. Horrell L, Goldsmith K, Tylee A, et al. One-day cognitive–behavioural therapy self-confidence workshops for people with depression: Randomised controlled trial. *Br J Psychiatry* 2014; **204**(3): 222-33.

30. Patel V, Weobong B, Weiss HA, et al. The Healthy Activity Program (HAP), a lay counsellor-delivered brief psychological treatment for severe depression, in primary care in India: a randomised controlled trial. *Lancet* 2017; **389**:176-85.

For peer review only - http://bmjopen.bmj.com/site/about/guidelines.xhtml

31. Nadkarni A, Weobong B, Weiss HA, et al. Counselling for Alcohol Problems (CAP), a lay counsellor-delivered brief psychological treatment for harmful drinking in men, in primary care in India: a randomised controlled trial. *Lancet* 2017; **389**:186-95.

32. Chibanda D, Weiss HA, Verhey R, et al. Effect of a Primary Care–Based Psychological Intervention on Symptoms of Common Mental Disorders in Zimbabwe: A Randomized Clinical Trial. *JAMA.* 2016**;316**(24):2618–26.

33. Watkins KE, Hunter SB, Hepner KA, et al. An effectiveness trial of group cognitive behavioral therapy for patients with persistent depressive symptoms in substance abuse treatment. *Arch gen psychiatry.* 2011;**68**(6):577-84.

34. Muñoz, R. F., & Ying, Y. W. (1993). The Prevention of Depression: Research and Practice. Johns Hopkins University Press. (Re-issued, 2003).

35. Muñoz, R F (1984). The Depression Prevention Course. https://i4health.paloaltou.edu/manuals/prevention-depression.html

36. Muñoz RF, Ying YW, Bernal G, et al. Prevention of depression with primary care patients: A randomized controlled trial. *Am J Community Psychol* 1995; **23**:199-222.

37. Muñoz R F & Mendelson T. Toward evidence-based interventions for diverse populations: The San Francisco General Hospital prevention and treatment manuals. *J Consult Clin Psychol* 2005; **73**:790-9.

38. Muñoz R F, Le HN, Ghosh Ippen C, et al. Prevention of postpartum depression in low-income women: Development of the Mamás y Bebés/Mothers and Babies Course. *Cogn Behav Pract* 2007; **14**:70-83.

39. Brown JS, Elliott SA, Boardman J, et al. Can the effects of a 1-day CBT psychoeducational workshop on self-confidence be maintained after 2 years? A naturalistic study. *Depress Anxiety* 2008; **25**(7):632-40.

40. Craig P, Dieppe P, Macintyre S, et al. MRC Guidance: Developing and evaluating complex interventions: the new MRC guidance. *BMJ* 2008;**337**:a1655.

41. Lancaster GA, Dodd S, Williamson PR. Design and analysis of pilot studies: recommendations for good practice. *J Eval Clin Pract* 2004; 10(2):307-12.

42. Shih WJ, Ohman-Strickland PA, Lin Y. Analysis of pilot and early phase studies with small sample sizes. *Statistics in Medicine* 2004;**23**:1827–42.

43. EuroQol Group: EuroQol–A New Facility for the Measurement of Health-Related Quality of Life, Volume 16. Amsterdam, Netherlands: *Health policy* 1990:199–208.

For peer review only

For peer review only - http://bmjopen.bmj.com/site/about/guidelines.xhtml

44. Thornicroft G, Brewin C R, Wing J. (Eds.) Measuring mental health needs. 1992 London, England: Gaskell/Royal College of Psychiatrists.

45. Attkisson CC, Zwick R: The client satisfaction questionnaire. Psychometric properties and correlations with service utilization. *Eval Program Plan* 1982; **5**(3):233–7.

46. Moore GF, Audrey S, Barker M, et al. Process evaluation of complex interventions: Medical Research Council guidance. *BMJ* 2015;**350**:h1258.

47. Ritchie J, Spencer L. Qualitative data analysis for applied policy research in A.Bryman and R. G. Burgess [eds.] *Analyzing qualitative data*. 1994.

48. Strauss A, Corbin J. *Basics of qualitative research techniques and procedures for developing grounded theory.* 2nd ed. SAGE: Thousand Oaks, CA; 1998.

Author's contributions: Authors WW, PB, CK, PC, JB, AB, MS, NL and MH, were involved in the conception and design of the study. CK drafted the manuscript and all authors WW, PB, PC, JB, AB, MS, NL, RFM were involved in revising it and providing or checking intellectual content. All authors have had final approval of the work and agree to be accountable for all aspects of the work.

Funding statement: This work was supported by National Institute for Health Research, School for Primary Care Research (Funding Ref 355).

Trial Sponsor: The University of Manchester. FBMHethics@manchester.ac.uk

Competing interests statement: None declared

Acknowledgements: We would like to thank the members of the public and Manchester PRIMER group (Primary Care Research in Manchester Engagement Resource) which supports PPI (Patient and Public Involvement) involvement in research, for their input and feedback on the development of the funding application.

For peer review only - http://bmjopen.bmj.com/site/about/guidelines.xhtml

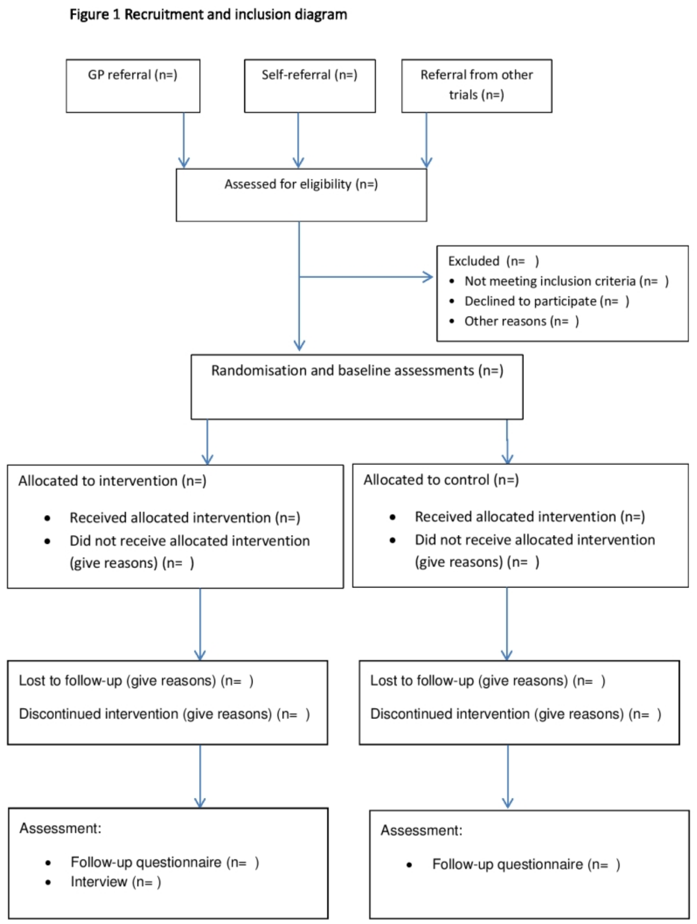

Figure 1 Recruitment and inclusion diagram

140x198mm (300 x 300 DPI)

BMJ Open

Preventing depression in the community by voluntary sector providers (PERSUADE): intervention development and protocol for a parallel randomized controlled feasibility trial.

Journal:	*BMJ Open*
Manuscript ID	bmjopen-2018-023791.R2
Article Type:	Protocol
Date Submitted by the Author:	28-Feb-2019

Complete List of Authors:	Kenning, Cassandra; University of Manchester, Division of Population Health, Health Services Research and Primary Care<br>Blakemore, Amy; University of Manchester,<br>Bower, Peter; University of Manchester, Division of Population Health, Health Services Research and Primary Care<br>Safari, Melina; University of Manchester, Division of Population Health, Health Services Research and Primary Care<br>Cuijpers, Pim; Vrije Universiteit Amsterdam<br>Brown, June S. L.; Kings College London<br>Hann , Mark ; University of Manchester, Centre for Biostatistics and Manchester Academic Health Science Centre<br>Lidbetter, Nicky; Self Help Services<br>Muñoz, Ricardo; Palo Alto University, Institute for International Internet Interventions for Health<br>Waheed, Waquas; University of Manchester, Division of Population Health, Health Services Research and Primary Care

**Primary Subject Heading**:	Public health
Secondary Subject Heading:	Qualitative research, Mental health
Keywords:	Depression & mood disorders < PSYCHIATRY, PUBLIC HEALTH, PREVENTIVE MEDICINE, MENTAL HEALTH

SCHOLARONE™ Manuscripts

Preventing depression in the community by voluntary sector providers (PERSUADE): intervention development and protocol for a parallel randomized controlled feasibility trial.

Cassandra Kenning. Division of Population Health, Health Services Research & Primary Care. The University of Manchester, Manchester UK.

Amy Blakemore. Division of Nursing, Social Work & Midwifery. The University of Manchester, Manchester UK.

Peter Bower. Division of Population Health, Health Services Research & Primary Care. The University of Manchester, Manchester UK.

Melina Safari. Division of Population Health, Health Services Research & Primary Care. The University of Manchester, Manchester UK.

Pim Cuijpers, Faculty of Behaviour and Movement Sciences. Vrije Universiteit, Amsterdam, The Netherlands.

June Brown, Department of Psychology. King's College London, London UK.

Mark Hann. Centre for Biostatistics and Manchester Academic Health Science Centre. The University of Manchester, Manchester UK.

Nicky Lidbetter, Self Help Services, Manchester UK.

Ricardo F. Muñoz, Institute for International Internet Interventions for Health. Palo Alto University. Palo Alto CA, US.

Waquas Waheed* Division of Population Health, Health Services Research & Primary Care. The University of Manchester, Manchester UK.

*corresponding author

Dr Waquas Waheed

The University of Manchester

5th Floor Williamson Building

Oxford Road

Manchester

M13 9PL

Waquas.waheed@manchester.ac.uk

Tel: 0161 306 7949

For peer review only - http://bmjopen.bmj.com/site/about/guidelines.xhtml

Word count: 4028

Abstract

Introduction

Depression is now the most common illness worldwide affecting more than 300 million people. Studies modelling the impact of depression interventions have shown that the burden of depression cannot be minimised by more than 35% with existing treatments. There is a need to develop better preventative interventions. The overall aim of this programme of work is to develop interventions to reduce depression symptom scores and prevent development of depression in people with sub-threshold depression. The objectives are to adapt a low intensity community based depression prevention intervention, establish the acceptability and uptake of this model, and to conduct a parallel randomized controlled feasibility trial to inform a full-scale trial.

Methods and analysis

Focus groups will be run with members of the public, voluntary sector providers, and researchers to inform the adaptation of an existing depression prevention course. Sixty four people with sub-threshold depression, as represented by a score of between 5 and 9 on the PHQ-9 depression measure, will be recruited to take part in the feasibility trial. Participants will be randomised equally to the intervention or usual care control groups. Participants in the intervention group will receive the new revised manual and attend a one-day workshop delivered by voluntary sector service providers. Outcome measures will be completed three months after baseline. Quantitative data on recruitment, randomisation, attendance, retention, questionnaire completion rates will be collected. Primary analyses will be descriptive and a process evaluation conducted to assess the processes involved in implementing the intervention. Interviews will be conducted to explore acceptability and framework analysis will be used to analyse the data.

Ethics and dissemination

The study has been reviewed and approved by NHS Research and Ethics Committee: NW-Greater Manchester East. The results will be actively disseminated through peer-reviewed journals, conference presentations, social media, the internet and community engagement activities.

Trial registration: ISRCTN23278208

Key words: sub-threshold, depression, prevention, protocol, feasibility, .

For peer review only - http://bmjopen.bmj.com/site/about/guidelines.xhtml

Strengths and limitations of this study

- Adapting the intervention to a workbook and one-day workshop will reduce costs, make it more accessible (lower burden) to participants, and move the intervention from primary care to voluntary sector organisations.
- Recruiting people with sub-threshold depression can be challenging and so the study will evaluate a number of different recruitment strategies and assess them to see how much time each takes and the associated yield.
- We are engaging substantively with members of the public as well as with voluntary sector services and charities to ensure the intervention is both feasible and acceptable.
- This is a feasibility trial and is not powered to show effectiveness of the intervention.

For peer review only

For peer review only - http://bmjopen.bmj.com/site/about/guidelines.xhtml

Introduction

Depression has high prevalence and incidence,[1] affects quality of life,[2] productivity, fulfilment of social roles, and increases mortality,[3, 4] leading to high service use and economic costs.[5] Depression is a major contributor to global disease burden, predicted to become the leading contributor in high-income countries by 2030.[6] Recent reports by the World Health Organisation (WHO), show that depression is the leading cause of disability worldwide, affecting more than 300 million people.[7] In a recent community cohort study of whites and British Pakistanis, prevalence of depression was 12.6%. At 6 months follow up 60.5% cases were persistently depressed and incidence of new cases was 17%.[8]

Modelling studies have shown that existing treatments cannot reduce the burden of depression by more than 35%, even under ideal circumstances.[9] Major depression can be prevented with current knowledge. [10,11] Prevention may offer an opportunity to reduce the disease burden of depression further.[12] A number of studies have examined the capability of prevention programmes to successfully reduce the incidence of depression.[13,14] This is important from a public health perspective as duration of the disorder is inversely related to outcome, so that by the time cases are recognised, they are harder to treat. Developing an intervention aimed at both prevention and early treatment could potentially reduce the overall burden of depression.[6]

NICE guidelines (CG 90 and 91) advocate case-finding by GP for depression in high risk groups with physical comorbidities, and those with past history of depression. They recommend offering anti-depressants or psychological interventions to people with moderate to severe depression.[15] In the UK access to psychological therapies for depression is delievered by IAPT teams (Improved Access to Psychological Therapies). According to the service guidelines they offer screening and then access to services for people scoring above 10 on the Patient Health Questionnaire (PHQ-9) screening tool. [16] A range of evidence-based psychological interventions are delivered according to a stepped-care model for those with mild-moderate and moderate-severe depression.[17]

Less attention has been paid to sub-threshold depression, defined as those who respond positively to case-finding questions on the PHQ-9 but do not have sufficient symptom scores on diagnostic criteria to screen positive for major depressive episode.[18] Sub-threshold depression is a major risk factor for progression and development of more severe depression[19,20] and has been shown to have similar consequences.[21-23]

Previous UK trials have generally included cases with above threshold depression and have not looked exclusively at people with sub-threshold depression. We could identify only two UK RCTs in the two published meta-analyses of 25 depression prevention trials.[24, 25] Both UK trials were aimed exclusively at pregnant women and published in 2000. An update of this meta-analysis included 34 RCTs with no further RCTs from UK.[26]

An intervention for sub-threshold depression needs to reduce the incidence of new onset depression but also improve outcome for those who do develop depression by reducing the length of the episode. GPs coordinating with voluntary sector organisations to deliver the intervention will potentially increase access for hard to reach groups.[27-29] Depression treatment studies using lay health workers to deliver cognitive behavioural interventions have already been found effective in settings with few professional mental health providers. [30-32] However, voluntary sector workers

For peer review only - http://bmjopen.bmj.com/site/about/guidelines.xhtml

will need to be adequately trained and supervised in delivering a patient-centred intervention according to a treatment manual. Substance abuse treatment counsellors have successfully administered a version of the depression prevention intervention to treat depression in individuals in substance abuse treatment.[33]

We need to understand user and provider views about prevention strategies in subthreshold depression and their applicability to UK health systems. There is uncertainty around the content, format, duration and use of voluntary sector workers to deliver such an intervention. We will aim to address these issues through involving users, providers, primary care staff and experts in this field and incorporating their opinions into the intervention and trial design.

The model we propose to use will be a psycho-educational intervention for prevention of depression in the form of a community based workshop to be delivered to people with sub-threshold depression. The proposed intervention will be adapted from the “Depression Prevention Course” and include evidence based CBT techniques, previously developed by Muñoz for preventing depression. The intervention is fully described in the book: The Prevention of Depression: Research and Practice [34] and an online version of the manual is freely available. [35] Many trials have already used versions of the manals from the depression prevention course and have shown efficacy in diverse populations. [36-38] The adapted intervention will be delivered via an accessible community workshop format as developed for depressed people by Brown (Self-confidence workshops).[28,29,39]

Research objectives

The eventual aim of our research programme is to reduce depression symptom scores and prevent development of depression in people with sub-threshold depression defined as PHQ score 5-9.[16] This feasibility trial will follow the MRC framework [40] for the development and evaluation of complex interventions, and has the following objectives:

1. To adapt a low intensity community based depression prevention intervention based on CBT model for adults with sub-threshold depression deliverable by voluntary sector organisations.

2. To establish the acceptability and uptake of this model by adults with sub-threshold depression.

3. To test the feasibility of conducting a successful trial of community based depression prevention intervention for adults with sub-threshold depression.

Methods and analysis

Patient and Public Involvement

Over a number of years we have conducted research in collaboration with community based voluntary organisations (Roby Church, Mind, Pakistani resource centre, Scaitcliffe Community Centre) in the North West. During past stakeholder events it had been discussed that interventions should be made available that prevent depression in vulnerable people.

For peer review only - http://bmjopen.bmj.com/site/about/guidelines.xhtml

During the preparation of the funding application we worked with voluntary sector organisations and providers (YARAN Community services, Self Help Services, Manchester) to inform the proposed intervention content, delivery and research design. We also made presentations to a lay public group and incorporated their suggestions into the proposal. YARAN Community services and Self Help Services, Manchester will continue to offer advice throughout the project. Phase 1 of the study involves community focus groups and at the end of the study community events will be used to feed back the results and to develop the proposal for a full trial for effectiveness and cost-effectiveness. Members of the public will be involved in the recruitment to the study by providing feedback on recruitment materials during the focus groups but will not be involved in the conduct of the study.

Qualitative interviews with participants in the intervention group will focus on their experiences of a, taking part in a study and b, all aspects of the intervention (manual and workshop) to explore acceptability, burden of the intervention and to further modify or adapt the intervention and trial design for a full-trial.

Phase 1- intervention development

Intervention and manual development: The ‘Depression Prevention Course’ developed by Muñoz[34,35] will be adapted for this study. Box 1 below shows key components of the original Depression Prevention Course.

Box 1- Depression Prevention Course

Originally based on Social Learning Theory

The intervention was developed using materials from the book ‘Control your Depression’ 1978

Combines three methods used to treat depression:

- Increasing pleasant activities
- Social skills training
- Cognitive approaches

Delivered by trained CBT therapist

8 group sessions delivered over 8 weeks

51 page ‘Outlines for participants’ manual (text only)

Focus groups: To tailor the intervention, focus groups will be held with users and providers.

A series of focus groups (3-5) will be run with a variety of groups including the general population, voluntary service providers from Self-help Manchester, and academic participants. The aim of the groups will be to get feedback on intervention content, delivery options, manual design, as well as recruitment methods. This will assist in the intervention adaptation and delivery, and also in refining

For peer review only - http://bmjopen.bmj.com/site/about/guidelines.xhtml

the recruitment methods and materials. As the different aspects are discussed the groups will be asked to try to reach a consensus on their feedback, for example when offered a range of images, which do they think are the most engaging or in the case of terminology do they prefer 'depression' or 'low mood' or 'feeling down' among other suggestions. The groups will be held at a location convenient to participants and last around 2 hours. Sessions will be audio recorded, with consent, and transcribed.

Prevent depression Intervention:

Once we have run the focus groups, and discussed the outcomes within the trial team, the original Muñoz manual will be redesigned as an accessible and easy to understand manual based intervention. To support the manual and guide people in its use, participants will attend a one day (or two half-day) workshop. The aim is that offering a one-off psychoeducational group will improve access for a larger group of people. Asking participants to commit to 8 weeks of intervention when they do not yet have a diagnosable condition requires substantial resources and time commitment from service providers and participants. This workshop approach has been effective in a number of studies.[27,28]

Phase 2- Feasibility trial

PERSUADE Protocol v2 (20/08/2017)

Design: A mixed methodsfeasibility trial and process evaluation to explore feasibility and acceptability of a depression prevention intervention.

Setting: Primary care and community, Greater Manchester UK.

Feasibility parameters: best recruitment methods to identify people with sub-threshold depression, participants willingness to be randomised, retention rates and outcome measure completion rates, acceptability of the intervention to both participants and facilitators, training of facilitators from voluntary sector services to deliver the intervention.

Inclusion criteria: Patients from primary care and the general community who are aged above 18 years and who score between 5 and 9 on the PHQ-9.[16]

Exclusion criteria:
Candidates will be asked if they have any diagnoses for:

- Depression (score higher than 9 on PHQ-9, or formal diagnosis of depression)
- Psychosis (self-report)
- Drug or alcohol use as primary diagnosis (self-report)
- Suicidal ideations (self-report)
- Non-English language speakers

Participant recruitment: As this is a feasibility trial, a formal sample size calculation was not undertaken. The aim is to recruit a total of 64 patients to be randomised with equal probability to

For peer review only - http://bmjopen.bmj.com/site/about/guidelines.xhtml

the intervention or usual care (i.e. 32 to each). This is broadly in line with current guidance on feasibility trials [41] and was considered sufficient to allow the formation of multiple groups for testing of the intervention, and to provide sufficiently robust estimates of recruitment timelines and rates of loss to follow up, within the limits of the available funding.

Considering the challenge of recruiting persons with sub-threshold depression, a number of different recruitment strategies will be tried and assessed to see how much time each takes and the associated yield.

Recruitment through GP practices:

- GPs to refer patients recently screened with the PHQ-9 who scored between 5 and 9. They will either give out the study information during the consultation or post the patient materials to them.
- Searches will be carried out on GP practice databases to see if it is feasible to identify patients who have concerns about their mood but who have not been referred on to IAPT (Improving Access to Psychological Therapies) services due to scores below 9 on the PHQ-9. Searches will be done by practice staff or CRN support staff to identify potential participants and mail out the study information.
- Research staff to offer study information and to screen patients with PHQ-9 in GP waiting rooms if they are interested in taking part.

Network support will be sought from Greater Manchester NIHR Clinical Research Network (CRN) to ensure that the research project is conducted within the proposed timeframe and recruitment targets are achieved. CRN will help with engaging primary care and liaising with GP at site.

Recruitment through advertising:

- Voluntary sector organisations (Scaitcliffe centre, YARAAN community, Self-help Services, Manchester) to direct self-referring patients by providing information about the study.
- Advertise in GP practice, library, community centres, citizens' advice bureau, Sure Start and places of worship.
- Advertise on dedicated Website, Facebook and Twitter.
- Advertise the study in local newspapers/radio, and on Eventbrite.

Recruitment through other trials:

- Invite participants who are screened out from any ongoing depression trials run under Clinical Research Network. For those trials that agree, once patients have been screened as ineligible from other depression trials due to a low score on PHQ-9, they will send the details of PERSUADE trial to ineligible patients.

Consent

For peer review only - http://bmjopen.bmj.com/site/about/guidelines.xhtml

Patients consenting to be contacted by the research team will be contacted over the phone or if needed, visited at home or a convenient location. They will be given a participant information sheet and have the study clearly explained to them, and will be given the opportunity to ask questions. The researcher will screen the participant for eligibility (as above) and if patients agree and meet the inclusion criteria they will be asked to sign and return the 'participant consent form' to enter the trial. Baseline assessments will then be self-completed or if assistance is needed, by telephone.

Confidentiality and data storage

All participants will be assigned a unique identification number which will be used for randomisation, data collection documents and analysis. Consent forms will be stored separately from participant data in locked filing cabinets. Publication of results will retain identification numbers to preserve anonymity, particulary where direct quotes are used. Only the primary research team will have access to the final trial data set.

Ineligible and non-recruited participants

In cases where either moderate to severe depression (scoring 10 or more on PHQ-9) or thoughts of current self-harm or suicide are expressed, potential participants will be referred back to their GP. The PHQ-9 includes the statement: "Thoughts that you would be better off dead or of hurting yourself in some way". If candidates score highly on this question (2 or 3) they will be advised to see there GP as soon as possible to discuss their mood.

Randomisation Participants will be randomised with equal probability to the CBT-based workshops or usual care (i.e. 32 to each). Computer generated block randomisation will be used. The allocation sequence will be set up by someone independent of the research team. Once a participant is randomised the trial research associate will be informed by email and they will then contact participants. Participants will be informed of the allocation by letter or telephone. As this is an active intervention they will not be blinded. Also as this is a feasibility trial and not a trial of efficacy the research associate/PhD student will not be blind to allocation.

Figure 1, below describes the process for the entry of participants into the trial and data collection points.

We will follow the predetermined Stop-Go criteria for definitive RCTs:

i. GO if 52 or more patients are recruited into the study (the target 64 minus 10%)
ii. STOP if <39 (60%) patients recruited into study
iii. STOP Intervention is unacceptable to participants

Intervention

Format and content of the intervention

For peer review only - http://bmjopen.bmj.com/site/about/guidelines.xhtml

We will train voluntary sector workers to facilitate community workshops that will last up to 8 hours and will be delivered either on a single day or be split over 2 days (evenings and weekends). Participants will attend one of these workshops. There will be no restrictions on the type of care participants can receive from other services.

Data on content, design and format of prevention interventions has already been extracted and will be published as a review, this will guide us in adapting the "Depression Prevention Course"[34] for this study. Motivational techniques will be incorporated in the beginning of the workshop to help engage participants, based on previous research and our own experience of conducting social groups. Group exercises, role-play demonstrations and discussions of case vignettes will be participatory to help maintain participant's interest and motivation throughout the workshop. Participants will receive a professionally designed manual, in colour and with graphics, that describes the workshop's program and all content. Workshops will be assessed as part of a process evaluation and fidelity of the delivery of the intervention will be rated. Using a template, the workshops will be assessed for adherence to the intervention. A record will also be made of the amount of engagement and participation in the groups. Length of the workshops and if participants remain for the entire session will also be recorded and reported. These data will be used to evaluate the feasibility of training voluntary sector staff to deliver the intervention in conjunction with qualitative interviews with the workshop facilitators. The outcome of this evaluation will be used to inform the training package for workshop facilitators in the main randomised controlled trial.

We anticipate that there will be low loss to follow-up as this is a single session intervention and we will be able to assess the feasibility of a larger study and estimate group means, standard deviations and percentages for key outcomes (to provide some input into the future sample size calculations).[41,42]

Usual care group

Participants randomised to the usual care arm will receive whatever care is available to them in the community or primary care. We will document the self-reported care received by the participants in the usual care arm at 3 months follow up. There will be no restrictions on the type of care these participants can receive.

Training of facilitators

Voluntary sector facilitators will be recruited from 'Self-help Services Manchester'. There are no exclusion criteria but facilitators need: Experience of working or volunteering in a community setting; Experience of working with groups; Knowledge of mental health problems and an interest in the area of mental health. We aim to recruit four facilitators to work in pairs to deliver the workshops. The volunteer facilitators will be trained by WW and AB to deliver the intervention in a group setting. All facilitators will attend a two-day training event held at the University, teaching them the basics of providing group CBT interventions (elements of behavioural activation and cognitive restructuring) and how to deliver the manual. The aim is for them to support the manual by guiding the participants in some of the techniques described, not to deliver therapy. We will provide ongoing supervision as all the workshops will be evaluated by the research team as part of a process evaluation.

For peer review only - http://bmjopen.bmj.com/site/about/guidelines.xhtml
For peer review only

Outcomes

The primary outcome for this feasibility trial will be recruitment rates. Screening questions,demographic information, baseline and follow-up assessments will be recorded for each participant. Data will also be recorded regarding intervention attendance and retention rates for the three month follow-up.

Quantitative outcomes

To explore outcomes and completion rates, quantitative measures including mood, function, participation, costs, and acceptability will be collected.

Baseline questionnaires will include:

- Depression (PHQ-9) a nine item self-report measure[16] PHQ-9 scores of 5, 10, 15, and 20 represent mild, moderate, moderately severe, and severe depression. A widely used screening measure for depression, it has displayed good internal reliability (Cronbach's α = 0.89).
- Health status (EuroQoL) EQ-5D-3L, measured on five dimensions on three-point scales. Is a widely established measure showing high levels of internal validity and reliability in diverse populations and in many languages. [43]
- Client Service Receipt Inventory (CSRI), is an adaptable healthcare utilisation measure which records services used by patients in the community, primary and secondary care. The measure asks patients to report the number of visits with different healthcare services and practitioners within a three month time-frame .[44]

Outcomes will be assessed at three months post randomisation,using the same baseline measures with the addition of Client Satisfaction Questionnaire (CSQ-8) an eight-item scale measuring satisfaction on a four-point scale. The measure reports excellent internal reliability (coefficient α = 0.93).[45] All participants will be asked to complete all outcome measures. The researcher doing the analysis will be blind to allocation and so all measures must be given to all participants. Quantitative data on recruitment, randomisation, attendance, retention, questionnaire completion rates will be collected.

Statistical analysis

Primary analyses will be descriptive. Recruitment and retention rates will be computed. The trial is not designed to detect differences in clinical outcomes and, as such, will focus on computing appropriate summary statistics (including a measure of variation) for each quantitative outcome. We will report on preliminary incidence figures in both groups by reporting if any participants screen positive for major depression at follow-up. This data, along with estimates of recruitment and attrition rates, will provide the information necessary to inform sample size calculations for a definitive trial.

Qualitative process evaluation

For peer review only - http://bmjopen.bmj.com/site/about/guidelines.xhtml

Acceptability of training to facilitators and of the intervention to participants will be explored using qualitative methods. Interviews will be used to explore the acceptability of the intervention, implementation, mechanisms, and context with a sample of the participants and the four facilitators as per MRC guidelines.[46] To achieve this we will conduct up to 15 semi-structured interviews (or until category saturation is achieved) with people receiving the intervention and after the primary outcomes have been assessed at 3 months follow up. We will explore participants' views on being identified as having 'sub-threshold depression' and what this meant to them, the acceptability of the study measures, acceptability of the group format (including content, duration of sessions, venues), relationships with other group members and facilitators; what has changed, if anything, since the intervention. We will interview the workshop facilitators to explore training, their experiences of delivering the intervention, and barriers and facilitators to working with this group of patients. Framework Analysis will be used to analyse the data.[47]

Analysis will be conducted according to the constant comparative method [48], whereby analysis is carried out concurrently with data collection so that emerging issues can be iteratively explored. Transcripts will be analysed independently and coded using qualitative data analysis software. Themes will be discussed by the research team to reach consensus and a coding framework that includes higher level themes and relevant data will be assembled. Each transcript will analysed individually and then in groups, with the facilitator transcripts analysed separately from the participant transcripts but with comparisons being made across datasets.

Discussion

According to the World Health Organisation (WHO), depression is now the most common illness worldwide affecting more than 300 million people.[7] The impact of depression and the scale of the problem make its treatment and management a key priority for health services. However, recent studies have shown that existing treatments cannot reduce the burden of depression by more than 35%, even under ideal circumstances.[9] Therefore as well as developing new treatments for depression it is essential that focus is placed on depression prevention.[12]

The long-term aim of this study is to adapt and tailor a fairly intensive existing intervention to patients with sub-threshold depression, making it easily accessible, cost-effective and sustainable in the community. To achieve these aims we are engaging substantively with members of the public as well as with voluntary sector services and charities to ensure the intervention is both feasible and acceptable. A pilot study will then be used to inform a definitive multi-centre trial to assess efficacy and cost-effectiveness.

The underlying concepts of the intervention have shown efficacy in a number of studies.[24,36,37,38] However, in its original form it requires substantial resources and time commitment from service providers and participants. By adapting the intervention to a workbook and one-day workshop this will reduce costs, make it more accessible (lower burden) to participants, and move the intervention from primary care to voluntary sector organisations. The aim will also be to reduce the likelihood of patients developing diagnosable depression and/or reduce episodes of future depression.

There are several key areas we need to ensure are considered during this feasibility trial: that the material is easy to understand at a range of reading levels and backgrounds; that the intervention

For peer review only - http://bmjopen.bmj.com/site/about/guidelines.xhtml

can be delivered by non-NHS, voluntary sector providers; that the training package produced is adequate for the job and gives facilitators the skills they will need; that we can effectively recruit people with sub-threshold depression to a trial; and that the outcome measures are the best measures for assessing a full trial.

Ethics and dissemination

The research was reviewed and received ethical approval from NHS REC NW-Greater Manchester East (ref:17/NW/0604) on 23/11/2017. Participants will be given a detailed participant information sheet (PIS) and will complete and sign a participant consent form prior to participation. Participants will be assigned a unique identification number to ensure anonymity and confidentiality of data collected.

The study already has a website as well as Facebook and Twitter accounts which we are using for recruitment and will also post study updates and results. As well as the protocol, we intend to publish further peer-reviewed articles and present at academic conferences. Engagement events will be held inviting participants, stakeholders and members of the public to summarise the results and get further feedback for the development of a full-scale trial for effectiveness and cost-effectiveness.

For peer review only

References

1. Waraich P, Goldner EM, Somers JM, et al. Prevalence and incidence studies of mood disorders: a systematic review of the literature. *Can J Psychiatry* 2004; **49**(2):124-138.

2. Saarni SI, Suvisaari J, Sintonen H, et al: Impact of psychiatric disorders on health-related quality of life: general population survey. *Br J Psychiatry* 2007; **190**:326–32.

3. Patel V, Thornicroft G. Packages of care for mental, neurological, and substance use disorders in low- and middle-income countries: PLoS Medicine series. *PLoS Med* 2009; **6**(10):e1000160.

4. Cuijpers P, Vogelzangs N, Twisk J, et al. Comprehensive meta-analysis of excess mortality in depression in the general community versus patients with specific illnesses. *Am J Psychiatry* 2014; **171**:453–62.

5. Berto P, D'Ilario D, Ruffo P, et al: Depression: cost-of-illness studies in the international literature: a review. *J Ment Health Policy Econ* 2000; **3**:3–10.

6. Mathers CD, Loncar D: Projections of global mortality and burden of disease from 2002 to 2030. *PLoS Med* 2006; **3**:e442.

7. World Health Organization. Depression Fact Sheet 22 March 2018. http://www.who.int/news-room/fact-sheets/detail/depression (accessed 24/08/2018)

8. Gater R, Tomenson B, Percival C, et al. Persistent depressive disorders and social stress in people of Pakistani origin and white Europeans in UK. *Soc Psych Psychiatr Epidemi* 2009; **44**(3):198-207.

9. Andrews G, Issakidis C, Sanderson K, et. al. Utilising survey data to inform public policy: comparison of the cost-effectiveness of treatment of ten mental disorders. *BJP* 2004; **184**:526–33.

10. Muñoz RF, Beardslee WR, & Leykin Y. Major depression can be prevented. *American Psychologist* 2012; **67:**285-95.

11. Muñoz, R.F., Schueller, S.M., Barrera, A.Z., Le, H.N., & Torres, L. (2014). Major depression can be prevented: Implications for research and practice. In I.H. Gotlib & C.L. Hammen (Eds.), *Handbook of depression*-Third edition (pp. 471-491). New York: The Guilford Press.

12. Cuijpers P, Beekman AT, Reynolds CF 3rd. Preventing depression: a global priority. *JAMA* 2012; **307**(10):1033-4.

13. Cuijpers P, van Straten A, Smit F, et. al. Preventing the onset of depressive disorders: a meta-analytic review of psychological interventions. *Am J Psychiatry* 2008; **165**(10):1272-80.

14. Muñoz, R. F., Cuijpers, P., Smit, F., Barrera, A.Z., & Leykin, Y. (2010). Prevention of major depression. Annual Review of Clinical Psychology, 6, 181-212.

15. NICE: The Treatment and Management of Depression in Adults. London: NICE; 2009.

For peer review only - http://bmjopen.bmj.com/site/about/guidelines.xhtml

16. Kroenke K, Spitzer RL, Williams JBW. The PHQ-9 - Validity of a brief depression severity measure. J Gen Intern Med 2001; 16(9):606–13.

17. National Collaborating Centre for Mental Health. The Improving Access to Psychological Therapies Manual. V1, June 2018. NHS England.

18. Baldwin R, Anderson D, Black S, et al. Guideline for the management of late-life depression in primary care. *Int J Geriatr Psychiatry* 2003;**18**(9):829-38.

19. Judd L, Akiskal H, Maser J, et al. A prospective 12-year study of subsyndromal and syndromal depressive symptoms in unipolar major depressive disorders. *Arch Gen Psychiatry* 1998; **55**(8):694-700.

20. Cuijpers P, Smit F. Subthreshold depression as a risk indicator for major depressive disorder: a systematic review of prospective studies. *Acta Psychiatr Scand* 2004; **109**:325-31.

21. Cuijpers P, De Graaf R, Van Dorsselaer S. Minor depression: risk profiles, functional disability, health care use and risk of developing major depression. *J Affect Disord* 2004; **79**:71-9.

22. Cuijpers P, Smit F, Oostenbrink J, et al. Economic costs of minor depression: A population-based study. *Acta Psychiatr Scand* 2007; **115**:229-36.

23. Cuijpers P, Vogelzangs N, Twisk J, et al. Differential mortality rates in major and subthreshold depression? A meta-analysis of studies that measured both. *Br J Psychiatry* 2013; **202**:22-7.

24. Cuijpers P, Muñoz RF, Clarke GN, et al. Psychoeducational treatment and prevention of depression: the "Coping with Depression" course 30 years later. *Clin Psychol Rev* 2009;**29**(5):449-58.

25. Cuijpers P, van Straten A, Smit F, et al. Preventing the onset of depressive disorders: a meta-analytic review of psychological interventions. *Am J Psychiatry* 2008;**165**(10):1272-80.

26. van Zoonen K, Buntrock C, Ebert DD, et al. Preventing the onset of major depressive disorder: a meta-analytic review of interventions. *Int J Epidemiol* 2014;**43**(2):318-29.

27. White J, Keenan M, Brooks N. Stress control: a controlled comparative investigation of large group therapy for generalized anxiety disorder. *Behav Psychother* 1992; **20**: 97–114.

28. Brown JS, Elliott SA, Boardman J, et al. Meeting the unmet need for depression services with psycho-educational self-confidence workshops: preliminary report. *Br J Psychiatry* 2004;**185**:511-5.

29. Horrell L, Goldsmith K, Tylee A, et al. One-day cognitive–behavioural therapy self-confidence workshops for people with depression: Randomised controlled trial. *Br J Psychiatry* 2014; **204**(3): 222-33.

30. Patel V, Weobong B, Weiss HA, et al. The Healthy Activity Program (HAP), a lay counsellor-delivered brief psychological treatment for severe depression, in primary care in India: a randomised controlled trial. *Lancet* 2017; **389**:176-85.

For peer review only

For peer review only - http://bmjopen.bmj.com/site/about/guidelines.xhtml

31. Nadkarni A, Weobong B, Weiss HA, et al. Counselling for Alcohol Problems (CAP), a lay counsellor-delivered brief psychological treatment for harmful drinking in men, in primary care in India: a randomised controlled trial. *Lancet* 2017; **389**:186-95.

32. Chibanda D, Weiss HA, Verhey R, et al. Effect of a Primary Care–Based Psychological Intervention on Symptoms of Common Mental Disorders in Zimbabwe: A Randomized Clinical Trial. *JAMA.* 2016**;316**(24):2618–26.

33. Watkins KE, Hunter SB, Hepner KA, et al. An effectiveness trial of group cognitive behavioral therapy for patients with persistent depressive symptoms in substance abuse treatment*. Arch gen psychiatry.* 2011;**68**(6):577-84.

34. Muñoz, R. F., & Ying, Y. W. (1993). The Prevention of Depression: Research and Practice. Johns Hopkins University Press. (Re-issued, 2003).

35. Muñoz, R F (1984). The Depression Prevention Course. https://i4health.paloaltou.edu/manuals/prevention-depression.html

36. Muñoz RF, Ying YW, Bernal G, et al. Prevention of depression with primary care patients: A randomized controlled trial. *Am J Community Psychol* 1995; **23**:199-222.

37. Muñoz R F & Mendelson T. Toward evidence-based interventions for diverse populations: The San Francisco General Hospital prevention and treatment manuals. *J Consult Clin Psychol* 2005; **73**:790-9.

38. Muñoz R F, Le HN, Ghosh Ippen C, et al. Prevention of postpartum depression in low-income women: Development of the Mamás y Bebés/Mothers and Babies Course. *Cogn Behav Pract* 2007; **14**:70-83.

39. Brown JS, Elliott SA, Boardman J, et al. Can the effects of a 1-day CBT psychoeducational workshop on self-confidence be maintained after 2 years? A naturalistic study. *Depress Anxiety* 2008; **25**(7):632-40.

40. Craig P, Dieppe P, Macintyre S, et al. MRC Guidance: Developing and evaluating complex interventions: the new MRC guidance. *BMJ* 2008;**337**:a1655.

41. Lancaster GA, Dodd S, Williamson PR. Design and analysis of pilot studies: recommendations for good practice. *J Eval Clin Pract* 2004; 10(2):307-12.

42. Shih WJ, Ohman-Strickland PA, Lin Y. Analysis of pilot and early phase studies with small sample sizes. *Statistics in Medicine* 2004;**23**:1827–42.

43. EuroQol Group: EuroQol–A New Facility for the Measurement of Health-Related Quality of Life, Volume 16. Amsterdam, Netherlands: *Health policy* 1990:199–208.

44. Thornicroft G, Brewin C R, Wing J. (Eds.) Measuring mental health needs. 1992 London, England: Gaskell/Royal College of Psychiatrists.

45. Attkisson CC, Zwick R: The client satisfaction questionnaire. Psychometric properties and correlations with service utilization. *Eval Program Plan* 1982; **5**(3):233–7.

For peer review only - http://bmjopen.bmj.com/site/about/guidelines.xhtml

46. Moore GF, Audrey S, Barker M, et al. Process evaluation of complex interventions: Medical Research Council guidance. *BMJ* 2015;**350**:h1258.

47. Ritchie J, Spencer L. Qualitative data analysis for applied policy research in A.Bryman and R. G. Burgess [eds.] *Analyzing qualitative data*. 1994.

48. Strauss A, Corbin J. *Basics of qualitative research techniques and procedures for developing grounded theory.* 2nd ed. SAGE: Thousand Oaks, CA; 1998.

Author's contributions: Authors WW, PB, CK, PC, JB, AB, MS, NL and MH, were involved in the conception and design of the study. CK drafted the manuscript and all authors WW, PB, PC, JB, AB, MS, NL, RFM were involved in revising it and providing or checking intellectual content. All authors have had final approval of the work and agree to be accountable for all aspects of the work.

Funding statement: This work was supported by National Institute for Health Research, School for Primary Care Research (Funding Ref 355).

Trial Sponsor: The University of Manchester. FBMHethics@manchester.ac.uk

Competing interests statement: None declared

Acknowledgements: We would like to thank the members of the public and Manchester PRIMER group (Primary Care Research in Manchester Engagement Resource) which supports PPI (Patient and Public Involvement) involvement in research, for their input and feedback on the development of the funding application.

Figure 1 Recruitment and inclusion diagram

For peer review only - http://bmjopen.bmj.com/site/about/guidelines.xhtml

For peer review only

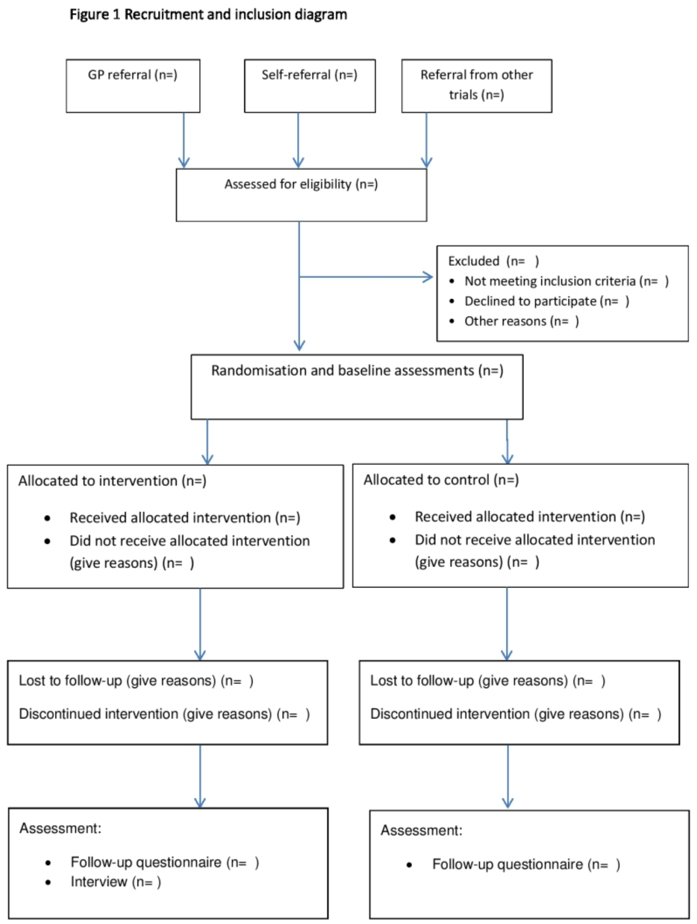

Figure 1 Recruitment and inclusion diagram

140x198mm (300 x 300 DPI)

SPIRIT

STANDARD PROTOCOL ITEMS: RECOMMENDATIONS FOR INTERVENTIONAL TRIALS

SPIRIT 2013 Checklist: Recommended items to address in a clinical trial protocol and related documents*

Section/item	Item No	Description	
**Administrative information**			
Title	1	Descriptive title identifying the study design, population, interventions, and, if applicable, trial acronym	P 1
Trial registration	2a	Trial identifier and registry name. If not yet registered, name of intended registry	P 2
	2b	All items from the World Health Organization Trial Registration Data Set	
Protocol version	3	Date and version identifier	P7
Funding	4	Sources and types of financial, material, and other support	P 14
Roles and responsibilities	5a	Names, affiliations, and roles of protocol contributors	P1, P14
	5b	Name and contact information for the trial sponsor	P 14
	5c	Role of study sponsor and funders, if any, in study design; collection, management, analysis, and interpretation of data; writing of the report; and the decision to submit the report for publication, including whether they will have ultimate authority over any of these activities	N/A
	5d	Composition, roles, and responsibilities of the coordinating centre, steering committee, endpoint adjudication committee, data management team, and other individuals or groups overseeing the trial, if applicable (see Item 21a for data monitoring committee)	NA
**Introduction**			
Background and rationale	6a	Description of research question and justification for undertaking the trial, including summary of relevant studies (published and unpublished) examining benefits and harms for each intervention	P4-5
	6b	Explanation for choice of comparators	P 9
Objectives	7	Specific objectives or hypotheses	P 5
Trial design	8	Description of trial design including type of trial (eg, parallel group, crossover, factorial, single group), allocation ratio, and framework (eg, superiority, equivalence, noninferiority, exploratory)	P 7

For peer review only

Methods: Participants, interventions, and outcomes

Study setting	9	Description of study settings (eg, community clinic, academic hospital) and list of countries where data will be collected. Reference to where list of study sites can be obtained	P7
Eligibility criteria	10	Inclusion and exclusion criteria for participants. If applicable, eligibility criteria for study centres and individuals who will perform the interventions (eg, surgeons, psychotherapists)	P 7, Fig.1
Interventions	11a	Interventions for each group with sufficient detail to allow replication, including how and when they will be administered	P9
	11b	Criteria for discontinuing or modifying allocated interventions for a given trial participant (eg, drug dose change in response to harms, participant request, or improving/worsening disease)	NA
	11c	Strategies to improve adherence to intervention protocols, and any procedures for monitoring adherence (eg, drug tablet return, laboratory tests)	P9-10
	11d	Relevant concomitant care and interventions that are permitted or prohibited during the trial	P9
Outcomes	12	Primary, secondary, and other outcomes, including the specific measurement variable (eg, systolic blood pressure), analysis metric (eg, change from baseline, final value, time to event), method of aggregation (eg, median, proportion), and time point for each outcome. Explanation of the clinical relevance of chosen efficacy and harm outcomes is strongly recommended	P10
Participant timeline	13	Time schedule of enrolment, interventions (including any run-ins and washouts), assessments, and visits for participants. A schematic diagram is highly recommended (see Figure)	P10, Fig.1
Sample size	14	Estimated number of participants needed to achieve study objectives and how it was determined, including clinical and statistical assumptions supporting any sample size calculations	P7, 10
Recruitment	15	Strategies for achieving adequate participant enrolment to reach target sample size	P7-8

Methods: Assignment of interventions (for controlled trials)

Allocation:

Sequence generation	16a	Method of generating the allocation sequence (eg, computer-generated random numbers), and list of any factors for stratification. To reduce predictability of a random sequence, details of any planned restriction (eg, blocking) should be provided in a separate document that is unavailable to those who enrol participants or assign interventions	P 8

For peer review only

Allocation concealment mechanism	16b	Mechanism of implementing the allocation sequence (eg, central telephone; sequentially numbered, opaque, sealed envelopes), describing any steps to conceal the sequence until interventions are assigned	P 8
Implementation	16c	Who will generate the allocation sequence, who will enrol participants, and who will assign participants to interventions	P 8
Blinding (masking)	17a	Who will be blinded after assignment to interventions (eg, trial participants, care providers, outcome assessors, data analysts), and how	P 8
	17b	If blinded, circumstances under which unblinding is permissible, and procedure for revealing a participant's allocated intervention during the trial	NA
**Methods: Data collection, management, and analysis**			
Data collection methods	18a	Plans for assessment and collection of outcome, baseline, and other trial data, including any related processes to promote data quality (eg, duplicate measurements, training of assessors) and a description of study instruments (eg, questionnaires, laboratory tests) along with their reliability and validity, if known. Reference to where data collection forms can be found, if not in the protocol	P10
	18b	Plans to promote participant retention and complete follow-up, including list of any outcome data to be collected for participants who discontinue or deviate from intervention protocols	P 10
Data management	19	Plans for data entry, coding, security, and storage, including any related processes to promote data quality (eg, double data entry; range checks for data values). Reference to where details of data management procedures can be found, if not in the protocol	P8, 10
Statistical methods	20a	Statistical methods for analysing primary and secondary outcomes. Reference to where other details of the statistical analysis plan can be found, if not in the protocol	P10
	20b	Methods for any additional analyses (eg, subgroup and adjusted analyses)	NA
	20c	Definition of analysis population relating to protocol non-adherence (eg, as randomised analysis), and any statistical methods to handle missing data (eg, multiple imputation)	NA
**Methods: Monitoring**			
Data monitoring	21a	Composition of data monitoring committee (DMC); summary of its role and reporting structure; statement of whether it is independent from the sponsor and competing interests; and reference to where further details about its charter can be found, if not in the protocol. Alternatively, an explanation of why a DMC is not needed	NA

For peer review only

	21b	Description of any interim analyses and stopping guidelines, including who will have access to these interim results and make the final decision to terminate the trial	NA
Harms	22	Plans for collecting, assessing, reporting, and managing solicited and spontaneously reported adverse events and other unintended effects of trial interventions or trial conduct	NA
Auditing	23	Frequency and procedures for auditing trial conduct, if any, and whether the process will be independent from investigators and the sponsor	NA
**Ethics and dissemination**			
Research ethics approval	24	Plans for seeking research ethics committee/institutional review board (REC/IRB) approval	P 2,11
Protocol amendments	25	Plans for communicating important protocol modifications (eg, changes to eligibility criteria, outcomes, analyses) to relevant parties (eg, investigators, REC/IRBs, trial participants, trial registries, journals, regulators)	
Consent or assent	26a	Who will obtain informed consent or assent from potential trial participants or authorised surrogates, and how (see Item 32)	P 8
	26b	Additional consent provisions for collection and use of participant data and biological specimens in ancillary studies, if applicable	NA
Confidentiality	27	How personal information about potential and enrolled participants will be collected, shared, and maintained in order to protect confidentiality before, during, and after the trial	P 8
Declaration of interests	28	Financial and other competing interests for principal investigators for the overall trial and each study site	P 14
Access to data	29	Statement of who will have access to the final trial dataset, and disclosure of contractual agreements that limit such access for investigators	P8
Ancillary and post-trial care	30	Provisions, if any, for ancillary and post-trial care, and for compensation to those who suffer harm from trial participation	NA
Dissemination policy	31a	Plans for investigators and sponsor to communicate trial results to participants, healthcare professionals, the public, and other relevant groups (eg, via publication, reporting in results databases, or other data sharing arrangements), including any publication restrictions	P2, 11
	31b	Authorship eligibility guidelines and any intended use of professional writers	NA
	31c	Plans, if any, for granting public access to the full protocol, participant-level dataset, and statistical code	NA

For peer review only

Appendices

Informed consent materials	32	Model consent form and other related documentation given to participants and authorised surrogates	NA
Biological specimens	33	Plans for collection, laboratory evaluation, and storage of biological specimens for genetic or molecular analysis in the current trial and for future use in ancillary studies, if applicable	NA

*It is strongly recommended that this checklist be read in conjunction with the SPIRIT 2013 Explanation & Elaboration for important clarification on the items. Amendments to the protocol should be tracked and dated. The SPIRIT checklist is copyrighted by the SPIRIT Group under the Creative Commons "Attribution-NonCommercial-NoDerivs 3.0 Unported" license.

For peer review only
